# Maternal supplementation with n-3 fatty acids affects placental lipid metabolism, inflammation, oxidative stress, the endocannabinoid system, and the neonate cytokine concentrations in dairy cows

**DOI:** 10.1186/s40104-024-01033-4

**Published:** 2024-05-21

**Authors:** Priscila dos Santos Silva, Gitit Kra, Yana Butenko, Jayasimha Rayalu Daddam, Yishai Levin, Maya Zachut

**Affiliations:** 1 Department of Ruminant Science, Institute of Animal Sciences, ARO Volcani Institute, Rishon LeZion, Israel; 2 Department of Animal Science, The Robert H. Smith Faculty of Agriculture, Food and Environment, The Hebrew University of Jerusalem, Rehovot, Israel; 3 Department of Animal Sciences, Michigan State University, East Lansing, MI USA; 4 The Nancy and Stephen Grand Israel National Center for Personalized Medicine, Weizmann Institute of Science, Rehovot, Israel

**Keywords:** Antioxidants, Dairy cows, Endocannabinoid system, Inflammation, Omega-3 fatty acids, Placenta

## Abstract

**Background:**

The placenta plays a crucial role in supporting and influencing fetal development. We compared the effects of prepartum supplementation with omega-3 (n-3) fatty acid (FA) sources, flaxseed oil (FLX) and fish oil (FO), on the expression of genes and proteins related to lipid metabolism, inflammation, oxidative stress, and the endocannabinoid system (ECS) in the expelled placenta, as well as on FA profile and inflammatory response of neonates. Late-pregnant Holstein dairy cows were supplemented with saturated fat (CTL), FLX, or FO. Placental cotyledons (*n* = 5) were collected immediately after expulsion, and extracted RNA and proteins were analyzed by RT-PCR and proteomic analysis. Neonatal blood was assessed for FA composition and concentrations of inflammatory markers.

**Results:**

FO increased the gene expression of fatty acid binding protein 4 (*FABP4*), interleukin 10 (*IL-10*), catalase (*CAT*), cannabinoid receptor 1 (*CNR1*), and cannabinoid receptor 2 (*CNR2*) compared with CTL placenta. Gene expression of ECS-enzyme FA-amide hydrolase (*FAAH*) was lower in FLX and FO than in CTL. Proteomic analysis identified 3,974 proteins; of these, 51–59 were differentially abundant between treatments (*P* ≤ 0.05, |fold change| ≥ 1.5). Top canonical pathways enriched in FLX vs. CTL and in FO vs. CTL were triglyceride metabolism and inflammatory processes. Both n-3 FA increased the placental abundance of FA binding proteins (FABPs) 3 and 7. The abundance of CNR1 cannabinoid-receptor-interacting-protein-1 (CNRIP1) was reduced in FO vs. FLX. *In silico* modeling affirmed that bovine FABPs bind to endocannabinoids. The FLX increased the abundance of inflammatory CD44-antigen and secreted-phosphoprotein-1, whereas prostaglandin-endoperoxide synthase 2 was decreased in FO vs. CTL placenta. Maternal FO enriched neonatal plasma with n-3 FAs, and both FLX and FO reduced interleukin-6 concentrations compared with CTL.

**Conclusion:**

Maternal n-3 FA from FLX and FO differentially affected the bovine placenta; both enhanced lipid metabolism and modulated oxidative stress, however, FO increased some transcriptional ECS components, possibly related to the increased FABPs. Maternal FO induced a unique balance of pro- and anti-inflammatory components in the placenta. Taken together, different sources of n-3 FA during late pregnancy enhanced placental immune and metabolic processes, which may affect the neonatal immune system.

**Supplementary Information:**

The online version contains supplementary material available at 10.1186/s40104-024-01033-4.

## Background

In all pregnant mammals, the developing fetus requires substantial amounts of fatty acids (FAs) during late gestation to support rapid cellular growth and activity [1]. Generally, omega-3 FAs (n-3 FAs) have anti-inflammatory, pro-resolving, and anti-oxidative properties [2–4]. Furthermore, n-3 FAs play an essential role in cellular metabolism, energy storage, and maintenance of homeostasis. The n-3 FAs influence the abundance of lipid mediators [5, 6] and can alter the cell membrane composition [7]. The FA composition delivered to the fetus is largely determined by the maternal circulating levels, and the placenta was shown to preferentially transfer physiologically important long-chain polyunsaturated FA (LCPUFA), especially n-3 FAs [2]. The transfer of nutrients, including lipids, from mother to fetus, is influenced by multiple factors [8, 9]. The bovine placenta features chorionic villi that are arranged in a cotyledonary manner, generating a complex architecture of blood vessels. Based on the maternal-fetal blood communication and the cell composition, the bovine placenta is classified as epitheliochorial/synepitheliochorial. It is composed of trinucleate feto-maternal hybrid cells, which form multiple cell layers that completely separate the maternal and fetal vascular systems, thus limiting their communication [10–13]. Moreover, FA transfer between the maternal and the fetal interface depends on factors such as the size of the molecule, and the method of transfer, depending on whether it is via diffusion or some form of active or facilitated transport [10, 14]. Additionally, the expression of the FA binding proteins (FABPs) and FA transport proteins (FATPs), which have high affinities with arachidonic acid (AA; C20:4n-6) and docosahexaenoic acid (DHA; C22:6n-3), facilitate the transport of FAs from the maternal bloodstream to the fetal circulation [9, 15, 16].

During late-gestation and towards calving, the metabolic activity of placental cells is notably high, producing various growth factors and forming reactive oxygen species (ROS), as well as secreting inflammatory molecules, which play an important role both in promoting placental development and in detaching the placental maternal-fetal unit [17–19]. However, adverse conditions, such as nutritional stress, can lead to an increased release of inflammatory cytokines and the production of ROS, which consequently have a negative impact on placental functionality. This can also be induced by inadequate placental vascularization, as well as a reduction in the conversion ratios of LCPUFA and the supply of essential nutrients needed to ensure the proper development of organs and the health of the fetus [1, 20–22]. Specifically, within the placenta, n-3 FAs modulate energy metabolism and improve placental angiogenesis and fetal growth [23].

Supplementation with n-3 FAs can also modulate the activation of the endocannabinoid system (ECS) [24, 25] by reducing the omega-6 (n-6) FAs to the n-3 FA ratio in the diet [26]. The ECS, which consists of the endocannabinoid ligands, cannabinoid receptors, and enzymes that synthesize and degrade the endocannabinoids, is involved in regulating energy metabolism and immune function [27]. The endocannabinoids anandamide (AEA) and 2-arachidonoylglycerol (2-AG) are synthesized from AA, and are hydrolyzed back to AA, ethanolamine, and glycerol by the enzymes fatty acid amide hydrolase (FAAH) and monoglyceride lipase (MGLL), respectively [28, 29]. In our previous studies on peripartum dairy cows, we described the effects of supplementing various sources of n-3 FAs, such as flaxseed oil (FLX) and fish oil (FO), on the ECS and immune function in the cow. We observed that peripartum n-3 FA supplementation reduces the gene expression of the ECS components in white blood cells, liver, and the adipose tissue, lowered the expression of the molecule linked to signalization of the inflammatory pathway in white blood cells such as nuclear factor kappa B (*NFκB*), and decreased the blood percentage of immune cells that are active during the inflammatory process, including CD25^+^ T-regulatory, when compared with controls postpartum [24, 30, 31]. Thus, supplementation with n-3 FAs affected the immune response and the ECS components in peripartum dairy cows.

Recently, maternal n-3 FAs and methionine supplementation in ewes [32] were shown to decrease the gene expression of FA binding protein 4 (*FABP4*) in placental cotyledons, suggesting that maternal n-3 FAs affect placental lipid transfer. However, currently, there is no information on the effects of n-3 FAs on the placenta of dairy cows. Our objective was to investigate the effect of n-3 FA supplementation on lipid metabolism, inflammatory response, oxidative stress, and the ECS components in the placenta of dairy cows at calving as well as the effect of the cytokine concentration on the plasma of the neonates. To this end, we compared two sources of n-3 FAs: FLX and FO. FLX is a source of alpha-linolenic acid (ALA; C18:3n-3), which, within the body, can undergo limited conversion to form both DHA and eicosapentaenoic acid (EPA; C20:5n-3) [33, 34], whereas FO contains DHA and EPA [35]. We hypothesized that supplementation of peripartum dairy cows with n-3 FAs, rich in either EPA, DHA (FO), or in their precursor ALA (FLX), would modulate lipid metabolism by improving the transfer of n-3 FAs through the placenta to the fetus, reduce the ECS components in the placenta, and induce anti-inflammatory and anti-oxidative effects. We hypothesized that these changes may be beneficial for the placental function and may improve the inflammatory response in neonates.

## Methods

### Animals, treatments and experimental procedures

The experimental protocol for the study was approved by the Volcani Center Animal Care Committee (approval number IL 797/18), and it was performed in accordance with the relevant guidelines and regulations. The experiment was conducted at the Volcani Center experimental farm in Rishon Lezion, Israel. A detailed description of this experiment was provided in [30]. Briefly, 42 multiparous Holstein dairy cows, with a mean parity of 3.8 ± 1.4 (mean ± standard deviation), participated in the study during the winter season (November 2018–March 2019). The average temperature-humidity index (THI) was 61 ± 8, indicating conditions well within the thermoneutral zone for dairy cows [36]. The cows were group-housed from d 257 of pregnancy to d 60 postpartum in a shaded loose pen that was equipped with a real-time electronic individual feeding system. Each feeding station included an individual identification system (ID tag; SAE Afikim, Kibbutz Afikim, IL) that allowed each cow to enter a specific feeding station only and automatically recorded each meal. The cows were stratified according to milk yields during the first 60 d of the previous lactation, body weight at drying off, and parity. The prepartum dietary treatments began at d 257 of pregnancy as follows: (i) control group (CTL; *n* = 14), fed a basal dry cow diet supplemented with encapsulated saturated fatty acid (SFA) at 240 g/d/cow; (ii) FLX (*n* = 14), fed a basal diet supplemented at 300 g/d/cow, with encapsulated flaxseed oil providing ALA at 56.1 g/d; and (iii) FO (*n* = 14), fed a basal diet supplemented at 300 g/d/cow with encapsulated fish oil providing EPA at 5.8 g/d and DHA at 4.3 g/d. The total fat content of the CTL supplement was 99%, compared to 80% in the FLX and FO supplements; therefore, the amounts of the supplements differed between the groups in order to maintain similar fat contents in all diets. The fat supplements were specially prepared and supplied by SILA (Venice, Italy). The ingredients and the chemical compositions of the rations, as well as the profile of the main FAs in the supplements were previously presented in [30, 37]. The neonate calves were separated from their mothers at calving, and were provided individually with their mothers’ colostrum immediately after blood samples were collected post-calving. A diagram describing the experimental design, the treatments, biological samples collected, and laboratory analyses are presented (Additional file 1: Fig. S1).

### Placenta collection

Placental tissues were collected immediately after the expulsion of the placenta postpartum. From each placenta, we dissected 4 or 5 samples (50 mg) of cotyledons that were stored immediately at −80 ºC for further RNA and protein extraction. In total, we collected 13 placenta samples during the experiment (3 CTL, 5 FLX, and 5 FO). Since we did not obtain sufficient placenta samples from the control cows, we collected 2 additional placenta samples from cows that were fed the basal dry cow diet (without SFA supplementation) during the winter season of the following year.

### Placental RNA extraction and transcript expression

We analyzed the gene expression in 5 placenta samples from each dietary treatment. Total RNA extraction of placental tissue was performed using an Animal tissue RNA purification kit (#25700, NORGEN BioTek Corp, Ontario, Canada). The RNA purity was assessed using a NanoDrop One Microvolume UV-Vis Spectrophotometer (Thermo Scientific, Shoham, IL) with a 260/280 ratio of above 1.85. First-strand cDNA was generated using the RevertAid First Strand cDNA Synthesis Kit (#K1622, Thermo Fisher Scientific, Vilnius, Lithuania). Quantitative detection of specific mRNA transcripts was carried out by real-time PCR using a CFX Duet Real-Time PCR System (Bio-Rad Laboratories, Inc., Rishon LeZion, IL) with the SYBR Green PowerTrack™ Master Mix (#A46109, Applied BioSystems, MA, USA) and analyzed using Bio-Rad CFX Maestro Software. In placenta tissues we examined the transcription levels of genes related to lipid metabolism, inflammation, oxidative response, and ECS. The list of primers is presented in Table 1. The reference genes β-Actin (*ACTB*), glyceraldehyde-3-phosphate dehydrogenase (*GAPDH*), and ubiquitously expressed prefoldin like chaperone (*UTX*) were examined. NormFinder software suggested *ACTB* and *UTX* as the most stable housekeeping genes for the placenta samples. The relative quantity of each gene was normalized to the average transcription levels of the reference genes (2^−^^∆∆Ct^ method) according to Livak et al. [38].

**Table 1:** Primer sequences used for qRT-PCR analysis of the placental tissue in this study

Gene^a^	GenBank accession No.	Sequence (5′→3′)^b^
*ACTB*	NM_173979.3	F: CTCTTCCAGCCTTCCTTCCT R: TAGAGGTCCTTGCGGATGTC
*CAT*	NM_001035386.2	F: ACATGGTCTGGGACTTCTGG R: TCAGTGCCTGTGTCCATCT
*CNR1*	NM_001242341.1	F: AAGCCCGCATGGACATTAGGTTAG R: AGCAGAGGGCCCCAGCAGAT
*CNR2*	NM_001192303.1	F: TCTTCGCCGGCATCATCTAC R: CATCCGGGCTATTCCAGACA
*FAAH*	XM_005197903.3	F: TTCCTGCCAAGCAACATACCT R: CACGAAATCACCTTTGAAGTTCTG
*FABP4*	NM_174314.2	F: TTCAAGCTGGGAGTCGAGTT R: TGTCCATTCCACTTCTGCAC
*FASN*	NM_001012669.1	F: ACCTCGTGAAGGCTGTGACTCA R: TGAGTCGAGGCCAAGGTCTGAA
*GAPDH*	NM_001040552.2	F: AGATGGTGAAGGTCGGAGTG R: GAAGGTCAATGAAGGGGTCA
*GPX3*	NM_001046088.2	F: CAGGGACAGGAGAAGTCGAA R: GCCAGCGTACTGCTTAAAGG
*IL1β*	NM_174093.1	F: CCATGGAGAAGCTGAGGAAC R: GGAGGACGTTTCGAAGATGA
*IL-6*	NM_001015617.1	F: CCCTCCAGGAACAGCTATGA R: GGGGTAGGGAAAGCAGAAGT
*IL6-R*	NM_001110785.1	F: GCTCTTTCTACGTATTGTCCCTGTGT R: GGGTCGGGCTGTAGGAGTTT
*IL-10*	NM_174088.1	F: CTGTATCCACTTGCCAACCA R: AAGCTGTGCAGTTGGTCCTT
*MGLL*	NM_001206681.1	F: GCAACCAGCTGCTCAACAC R: AGCGTCTTGTCCTGGCTCTT
*NAPEPLD*	NM_001015680.1	F: AGAGATCACAGCAGCGTTCCAT R: ACTCCAGCTTCTTCAGGGTCATC
*PPARG*	NM_181024.2	F: CCAAATATCGGTGGGAGTCG R: ACAGCGAAGGGCTCACTCTC
*SOD 1*	NM_174615.2	F: CGAGGCAAAGGGAGATACAG R: TCTCCAAACTGATGGACGTG
*SREBP1*	NM_001113302.1	F: CGACACCACCAGCATCAACCACG R: GCAGCCCATTCATCAGCCAGACC
*TLR-4*	NM_174198.6	F: GCTGTTTGACCAGTCTGATTGC R: GGGCTGAAGTAACAACAAGAGGAA
*TNFα*	NM_173966.3	F: CCATCAACAGCCCTCTGGTT R: GGGCTACCGGCTTGTTACTT
*UXT*	NM_001037471.2	F: TGTGGCCCTTGGATATGGTT R: GGTTGTCGCTGAGCTCTGTG

^a^*ACTB* Beta–Actin, *CAT* Catalase, *CNR1* Cannabinoid receptor 1, *CNR2* Cannabinoid receptor 2, *FAAH* Fatty acid amide hydrolase, *FABP4* Fatty acid binding protein 4, *FASN* Fatty acid synthase, *GAPDH* Glyceraldehyde-3-phosphate dehydrogenase, *GPX3* Glutathione peroxidase 3, *IL-1β* Interleukin 1-beta, *IL-6* Interleukin 6, *IL6-R* Interleukin-6 receptor, *IL-10* Interleukin 10, *MGLL* Monoglyceride lipase, *NAPEPLD* N-Acyl phosphatidylethanolamine phospholipase D, *PPARG* Peroxisome proliferator activated receptor gamma, *SOD1* Superoxide dismutase 1, *SREBP1* Sterol regulatory element binding transcription factor 1, *TLR4* Toll-like receptor 4, *TNFα* Tumor necrosis factor alpha, *UXT* Ubiquitously expressed prefoldin like chaperone

^b^*F* Forward, *R* Reverse

### Placental protein extraction

The placental samples (15 mg) were ground in a BeadBug homogenizer (D1030-E, Benchmark Scientific, Sayreville, NJ, USA) with two 0.5-mm glass beads (#11079105, BioSpec, Bartlesville, OK, USA) and 1 mL lysis buffer [5% (w/v) sodium dodecyl sulfate (SDS, #L3771-100) in 100 mmol/L Tris-HCl buffer containing 1% (v/v) phenylmethylsulfonyl fluoride (PMSF, #P7626), 1% phosphatase inhibitor (#P5726), and 1% protease inhibitor (#P8340), all from Sigma-Aldrich, St. Louis, MO, USA]. After centrifugation at 20,000 × *g* for 15 min at 4 °C, the soluble fractions were collected and the protein concentrations were measured using a bicinchoninic acid (BCA) standard assay (9470BCAstand, Cyanogen, Bologna, Italy), then snap-frozen and stored at −80 °C.

### Proteomic analysis

For proteomic analysis, we analyzed 3 placenta samples from each dietary treatment. From the controls, we selected the 3 samples that were available from the original experimental cohort, and in FLX and FO we randomly selected 3 samples from each treatment; overall, 9 placenta samples were subjected to mass spectrometry-based proteomic analysis. The samples were lysed with 5% SDS and digested with trypsin (#V528B, Promega, Madison, USA) using the S-trap method [39]. Samples were stored at −20 °C until further use.

#### LC/MS

Ultra Liquid Chromatography/Mass Spectrometry (ULC/MS)-grade solvents were used for all chromatographic steps. Each sample was loaded using nanoflow ultra performance liquid chromatography (UPLC) (nanoAcquity; Waters, Milford, MA, USA). The mobile phase consisted of (A) H_2_O + 0.1% formic acid (#06914144, Bio-Lab Ltd., Jerusalem, IL) and (B) acetonitrile (ACN, #0120502, Bio–Lab Ltd.) + 0.1% formic acid. The samples were desalted online using a Symmetry C18 trapping column (180 μm id, 20 mm length, 5 μm particle size; Waters). The peptides were then separated using a T3 HSS nano-column (75 μm id, 250 mm length, 1.8 μm particle size, Waters) at 0.35 µL/min. Peptides were eluted from the column into the mass spectrometer using the following gradient: 4%–29% B in 155 min, 29%–90% B in 5 min, maintained at 90% B for 5 min and then back to the initial conditions.

The Nano-UPLC was coupled online through a Nano-electrospray ionization mass spectrometry (NanoESI) emitter (10 μm tip; Fossil, Madrid, Span) to a Q Exactive HF mass spectrometer (Thermo Scientific, Massachusetts, USA). Data were acquired in data-dependent acquisition mode using the Top10 method. MS1 resolution was set to 120,000 (at 200 *m/z*), a mass range of 375–1,650 *m/z*, an automatic gain control (AGC) of 3e6, and the maximum injection time was set to 60 ms. MS2 was performed by isolation with the quadrupole, a width of 1.7 Th, 27 NCE, 15k resolution, an AGC target of 60 ms, and a dynamic exclusion of 45 s.

#### Proteomic data analysis

Raw data were processed with MetaMorpheus version 1.02 (available at https://github.com/smith-chem-wisc/MetaMorpheus). The following search settings were used: protease = trypsin, maximum missed cleavages = 2, minimum peptide length = 7, maximum peptide length = unspecified, initiator methionine behavior = Variable, fixed modifications = Carbamidomethyl on C, Carbamidomethyl on U, variable modifications = Oxidation on M, max mods per peptide = 2, max modification isoforms = 1,024, precursor mass tolerance = ± 5 parts per million (PPM), product mass tolerance = ± 20 PPM, and report peptide-spectrum match (PSM) ambiguity = True. The combined search database contained 37,704 non-decoy protein entries including 388 contaminant sequences. The proteins were quantified using the FlashLFQ method [40], embedded in MetaMorpheus. The quantitative comparisons were calculated using Perseus v1.6.2.3. Student’s *t*-test, after logarithmic transformation, was used to identify significant differences across the biological replica. Fold change (FC) was calculated based on the ratio of the geometric means of the case versus the control samples. Principal component analysis (PCA) revealed that one CTL sample was an outlier; therefore it was excluded from further analysis.

#### Bioinformatic analysis of proteomic data

Proteins with *P* ≤ 0.05 and a |FC| ≥ 1.5 were defined as significantly differentially abundant proteins (DAPs). Only DAPs with ≥ 1 unique peptides were analyzed using QIAGEN Ingenuity^®^ Pathway Analysis (IPA) software (QIAGEN, Inc., https://digitalinsights.qiagen.com/IPA) to determine the most relevant pathways, functions, and networks altered by the dietary treatments. Volcano plots were plotted with Microsoft Excel and bubble plots were plotted using the SRplot free online platform [41].

### *In-silico* docking studies of bovine FABPs with endocannabinoids

The FABPs link lipid metabolism and the ECS, since they serve as transporters of endocannabinoids within cells [42]; therefore, we aimed to determine whether bovine heart-type fatty acid binding protein (FABP3), epidermal-type fatty acid-binding protein (FABP5), and brain-type fatty acid binding protein (FABP7) bind to endocannabinoids by *in silico* modeling. To this end, FABP3 (Accession number: P10790), FABP5 (Accession number: P55052), and FABP7 (Accession number: Q09139) sequences were collected from the UNIPROT database using the *Bos taurus* model for modeling the protein structures. The endocannabinoids 2-AG, AEA and docosahexaenoyl ethanolamide (DHEA) were docked to target proteins using GOLD 3.0.1 software, a genetic algorithm that uses a strategy that covers three genetic operators such as migrations, mutations, and crossovers [43]. The compounds that docked into the active site of the target proteins were thoroughly studied by molecular mechanics calculations. The most energetically favorable conformation of each compound was identified and selected after docking. Each compound’s individual binding poses were studied, and interactions with the protein were calculated. A detailed description of the FABPs’ structure and docking modeling is shown in Additional file 3.

### Blood samples collected from neonate calves, FA compositions in plasma and in placenta tissues and inflammatory ELISA analysis

Blood samples were collected from calves (*n* = 9 per treatment) immediately after calving, before colostrum offering. They were collected from the jugular vein into vacuum tubes containing lithium heparin (#BD367526, Becton Dickinson Systems, Cowley, UK). Plasma was separated following centrifugation at 1,500 × *g* for 20 min at 4 °C, and stored at −80 °C, pending analysis. The FA composition in the placenta and in the plasma of neonate calves was determined as described previously [44]. Briefly, the samples were saponified in a mixture of 60% potassium hydroxide (KOH; #UN1813, Merck, Darmstadt, DE) and ethanol (#UN1170, Bio-Lab Ltd., Jerusalem, IL), extracted with petroleum ether (#UN1268, Bio-Lab Ltd.), and methylated with 5% (v/v) sulfuric acid (#UN1830, Bio-Lab Ltd.) in methanol (#UN1230, Bio-Lab Ltd.). FA methyl esters were analyzed using a 7890N gas chromatograph (Agilent Technologies, Santa Clara, CA) equipped with a DB-23 capillary column (60 m × 0.25 mm × 0.25 μm; Agilent Technologies) and a flame ionization detector. The initial temperature of the column was set at 130 °C, which was increased by 6.5 °C/min to 170 °C, and then by 2.75 °C/min to 215 °C, and held at 215 °C for 18 min. Then, the temperature was increased to 230 °C at 40 °C/min for the remainder of the analysis. The carrier gas was hydrogen, flowing at a linear velocity of 1.6 mL/min; the injection volume was 1 µL.

Plasma interleukin 2 (IL-2) and interleukin 6 (IL-6) concentrations were determined using Bovine Duoset ELISA kits (#DY2465 and #DY8190, respectively; R&D Systems, Inc., Minneapolis, MN, USA). Plasma haptoglobin (HP) concentrations were examined using a haptoglobin bovine ELISA kit (#E-10HPT, ICL, Portland, OR, USA).

### Statistical analysis

The placenta gene expression levels and the plasma variables from calves (IL-2, IL-6, and HP), as well as the FA composition from placenta or plasma were analyzed using a generalized linear model (GLM), using the following model: *Y*_*ijk*_ = *µ* + *T*_*i*_ + *C(T)*_*ij*_ + *E*_*ijk*_, where *Y*_*ijk*_ = dependent variable, *µ* = overall mean, *T*_*i*_ = treatment effect (*i* = CTL, FLX, or FO), *C(T)*_*ij*_ = cow or calf *j* nested in treatment *i*, and *E*_*ijk*_ = random residual. The data were analyzed using 9.4 Statistical Analysis System software after verifying the normality via the Shapiro-Wilk PROC single variable residual. The data were shown as the mean ± standard error of the mean (SEM) and declared significant when it reached *P* ≤ 0.05, and a tendency at 0.05 < *P* < 0.10 by Tukey’s test. The percentages of long-chain n-3 FAs EPA (C20:5n-3), DHA (C22:6n-3) and their intermediate docosapentanoic acid (DPA, C22:5n-3) from the calves’ plasma were not normally distributed; therefore, we analyzed the frequency of the appearance of these FAs by PROC FREQ and they were declared significant when they reached *P* ≤ 0.05 by Fisher’s test.

## Results

### Effects of maternal n-3 FA supplementation on placental gene expression

**Fig. 1:**
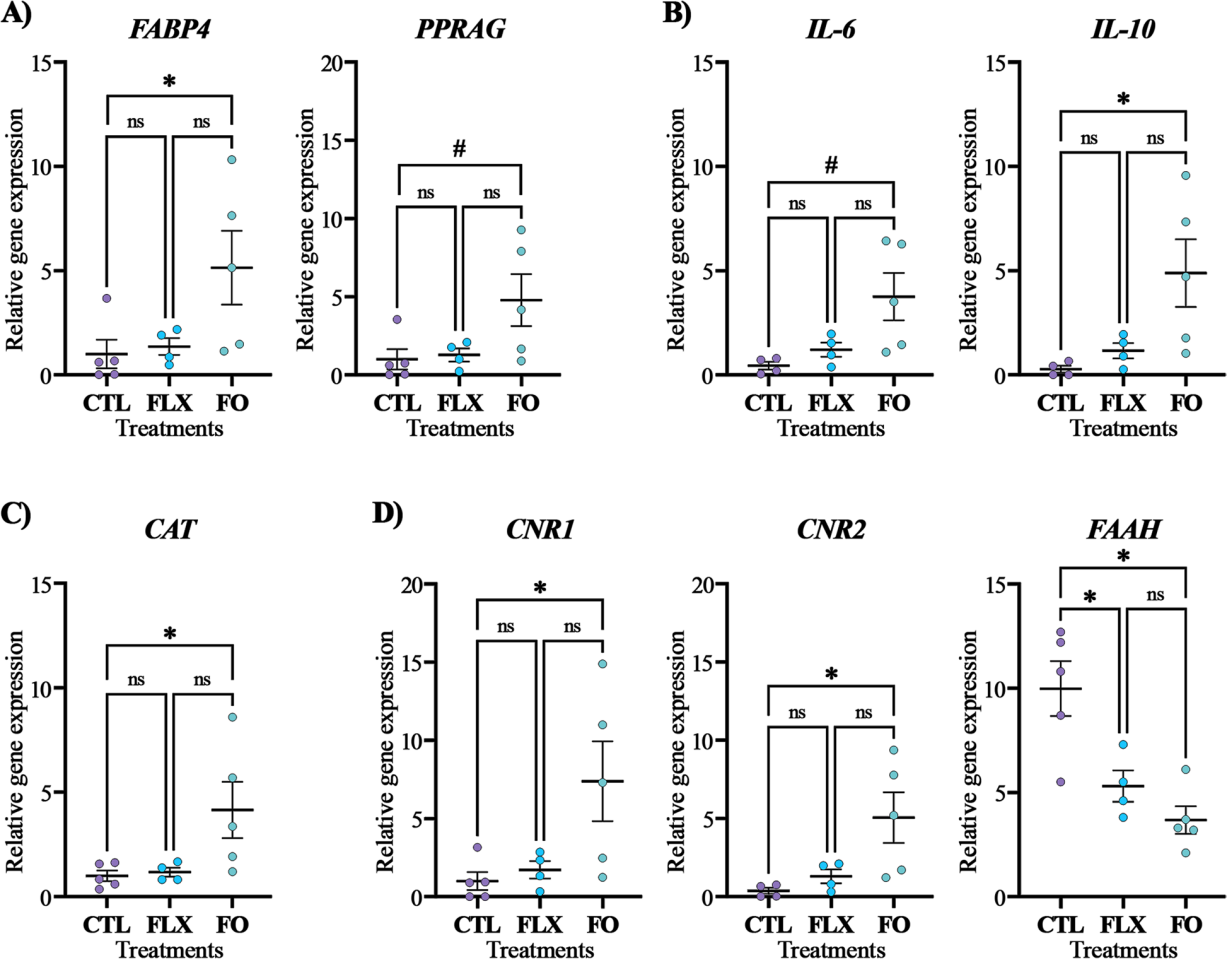
Expression levels of genes related to lipid metabolism, inflammation, oxidative stress, and ECS in placenta. (**A**) Lipid metabolism: *FABP4* Fatty acid binding protein 4, *PPARG* Peroxisome proliferator activated receptor gamma; (**B**) Inflammation: *IL-6* Interleukin 6, *IL-10* Interleukin 10; (**C**) Oxidative stress: *CAT* Catalase; and (**D**) ECS components: *CNR1* Cannabinoid receptor 1, *CNR2* Cannabinoid receptor 2, *FAAH* Fatty acid amide hydrolase. Dairy cows at d 257 of pregnancy were divided into 3 nutritional groups supplemented with (i) CTL – encapsulated saturated fat, (ii) FLX – encapsulated flaxseed oil providing ALA, or (iii) FO – encapsulated fish oil providing EPA and DHA. Data represent the mean ± SEM. * *P* ≤ 0.05 and # *P* < 0.1 when comparing CTL, FLX, and FO treatments by Tukey’s test

Several genes involved in lipid metabolism were examined; maternal FO supplementation increased the expression of the *FABP4* gene, which is involved in FA transfer in the placenta, by 5-fold more than CTL (*P* = 0.05; Fig. 1A), with no difference observed between CTL and FLX. Additionally, there was a tendency towards higher gene expression of peroxisome proliferator-activated-receptor gamma (*PPARG*; *P* = 0.06; Fig. 1A) in the FO placenta, compared with CTL. On the other hand, the average relative expressions of sterol regulatory element binding transcription factor 1 (*SREBP1*; *P =* 0.51) and fatty acid synthase (*FASN*; *P* = 0.31) were similar between treatments (data not shown).

In the analysis of the inflammation response genes, as shown in Fig. 1B, the FO placenta had higher gene expression of interleukin 10 (*IL-10*; *P* = 0.05) and a tendency for an increased expression of *IL-6* (*P =* 0.06) compared with CTL, with no difference observed between FLX and CTL. No significant differences between treatments were observed regarding the placental gene expressions of interleukin 1b (*IL-1b*; *P* = 0.28), interleukin-6 receptor (*IL6-R; P* = 0.32), the toll-like receptor 4 (*TLR4*; *P* = 0.44), and tumor necrosis factor alpha (*TNFα*; *P* = 0.86; data not shown).

Among the oxidative stress-related genes, the expression of catalase (*CAT*; *P* = 0.04) was significantly higher in FO than in CTL (Fig. 1C). There were no differences in glutathione peroxidase 3 (*GPX*3; *P =* 0.17) and superoxide dismutase 1 (*SOD1*; *P* = 0.45) expressions between treatments (data not shown).

Furthermore, maternal FO supplementation led to a significant increase in the placental expression of the ECS-related genes cannabinoid receptor 1 (*CNR1; P =* 0.03) and cannabinoid receptor 2 (*CNR2*; *P =* 0.04) compared with CTL (Fig. 1D). Both n-3 FA treatments (FLX and FO) significantly decreased the placental gene expression of the ECS enzyme *FAAH* (*P =* 0.002) compared with CTL (Fig. 1D). No difference was observed between treatments regarding the gene expressions of the ECS enzymes *MGLL* (*P =* 0.20) and N-acyl phosphatidylethanolamine phospholipase D (*NAPEPLD*; *P* = 0.57; data not shown).

### Effects of maternal n-3 FA supplementation on the placental proteome

**Fig. 2:**
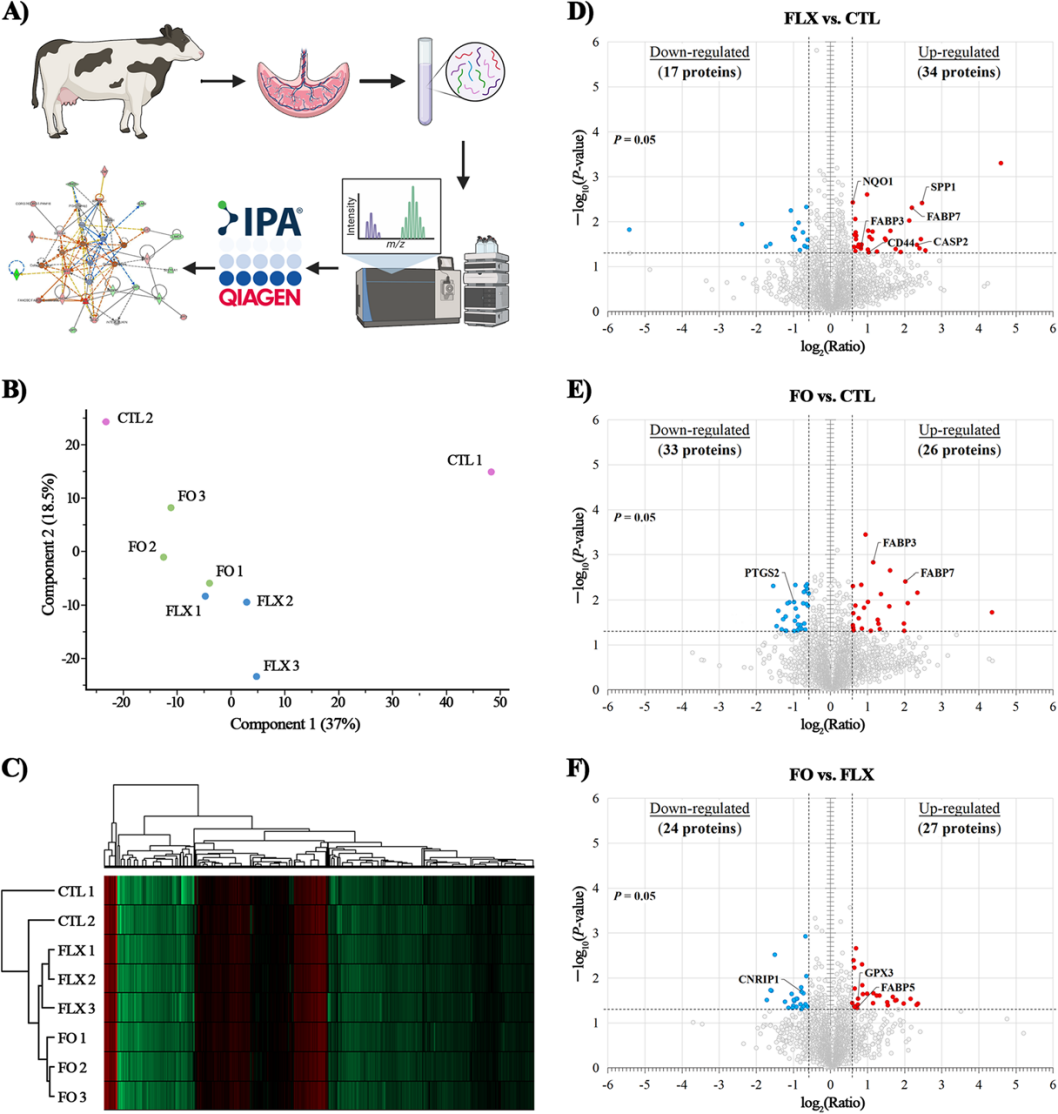
Proteomic analysis of the placenta of dairy cows supplemented pre-partum with n-3 FA. Placenta samples were collected immediately after delivery from dairy cows fed from d 257 of pregnancy with (i) CTL – encapsulated saturated fat, (ii) FLX – encapsulated flaxseed oil providing ALA, or (iii) FO – encapsulated fish oil providing EPA and DHA. (**A**) Work flow for the proteomic analysis; generated using BioRender.com. (**B**) Principal component analysis (PCA) of the placenta proteome; PCA analysis was used to assess the global integrity of the data and revealed that one CTL sample was an outlier, and therefore was excluded from further analysis; generated using Perseus v1.6.2.3. (**C**) Heat map analysis of the placental proteome: low peptide intensity is denoted in green, whereas high intensity is denoted in red. Each cow in the study was numbered and is represented in rows; generated using Perseus v2.0.11. (**D–F**) Volcano plot for the comparison between FLX vs. CTL (**D**), FO vs. CTL (**E**), and FO vs. FLX (**F**). *P-*value (≤ 0.05) is represented on the *Y*-axis and fold change (|FC| ≥ 1.5) is represented on the *X*-axis. Each dot represents one protein: red denotes up-regulated proteins, blue denotes down-regulated proteins

Overall, the proteomic analysis of the bovine placenta identified 3,974 proteins (Additional file 2). Of these, 51 proteins were differentially abundant (*P* ≤ 0.05 and |FC| ≥ 1.5) in FLX vs. CTL, whereas 59 were differential in FO vs. CTL, and 51 were differential in FO vs. FLX. Volcano plots illustrated that compared with CTL, the FLX treatment up-regulated 66.7% and down-regulated 33.3% of DAPs (Fig. 2D), whereas the FO treatment up-regulated 44.1% and down-regulated 55.9% of DAPs (Fig. 2E). Comparing DAPs in FO vs. FLX, 52.9% were up-regulated and 47.1% were down-regulated (Fig. 2F).

Of the DAPs, 2 proteins of the FABP family, FABP3 and FABP7, were significantly increased in FLX and FO compared with CTL. Interestingly, the proteomic data also revealed a higher abundance of the FABP5 (FC = 1.64, *P* = 0.04) in FO placenta vs. FLX. An additional protein implicated in the lipid metabolism was Caspase 2 (CASP2; FC = 5.04, *P* = 0.03), which was more abundant in FLX than in CTL. Furthermore, the n-3 FAs dietary treatments affected immune related proteins: FLX increased the abundance of CD44 antigen (CD44; FC = 2.04, *P* = 0.04) and secreted phosphoprotein 1 (SPP1; FC = 5.41, *P* = 0.02), whereas the prostaglandin-endoperoxide synthase 2 (PTGS2; FC = −1.97, *P* = 0.01) decreased in FO. The antioxidative protein NAD(P)H quinone dehydrogenase 1 (NQO1) increased in FLX, compared with CTL (FC = 1.51, *P* = 0.003), whereas GPX3 increased in FO, compared with FLX (FC = 1.67, *P* = 0.02). Another interesting protein that differed regarding FO vs. FLX was the ECS component CNR1-cannabinoid receptor-interacting protein 1 (CNRIP1), which was reduced in FO vs. FLX placenta (FC = −1.72, *P* = 0.02).

### Top canonical pathways, functions and networks according to the differential placenta proteome

The DAPs (proteins with |FC| ≥ 1.5 and *P* ≤ 0.05) were analyzed using Qiagen’s Ingenuity^®^ Pathway Analysis to identify the most relevant pathways, functions, and networks affected by FLX or FO dietary treatments. The main functions identified for DAP are presented in Additional file 1: Fig. S2. The most prominent biological function that changed between the treatments was regarding the levels of proteins with enzymatic functions: approximately 40% of DAPs in each comparison. Interestingly, FO supplementation resulted in a marked increase in the proportion of kinases and phosphatases among the differentially abundant enzymes, compared with FLX. Another important functional category identified in all the analyses was that of the proteins involved in transcription and translation regulation (Additional file 1: Fig. S2).

#### The top canonical pathways

**Fig. 3:**
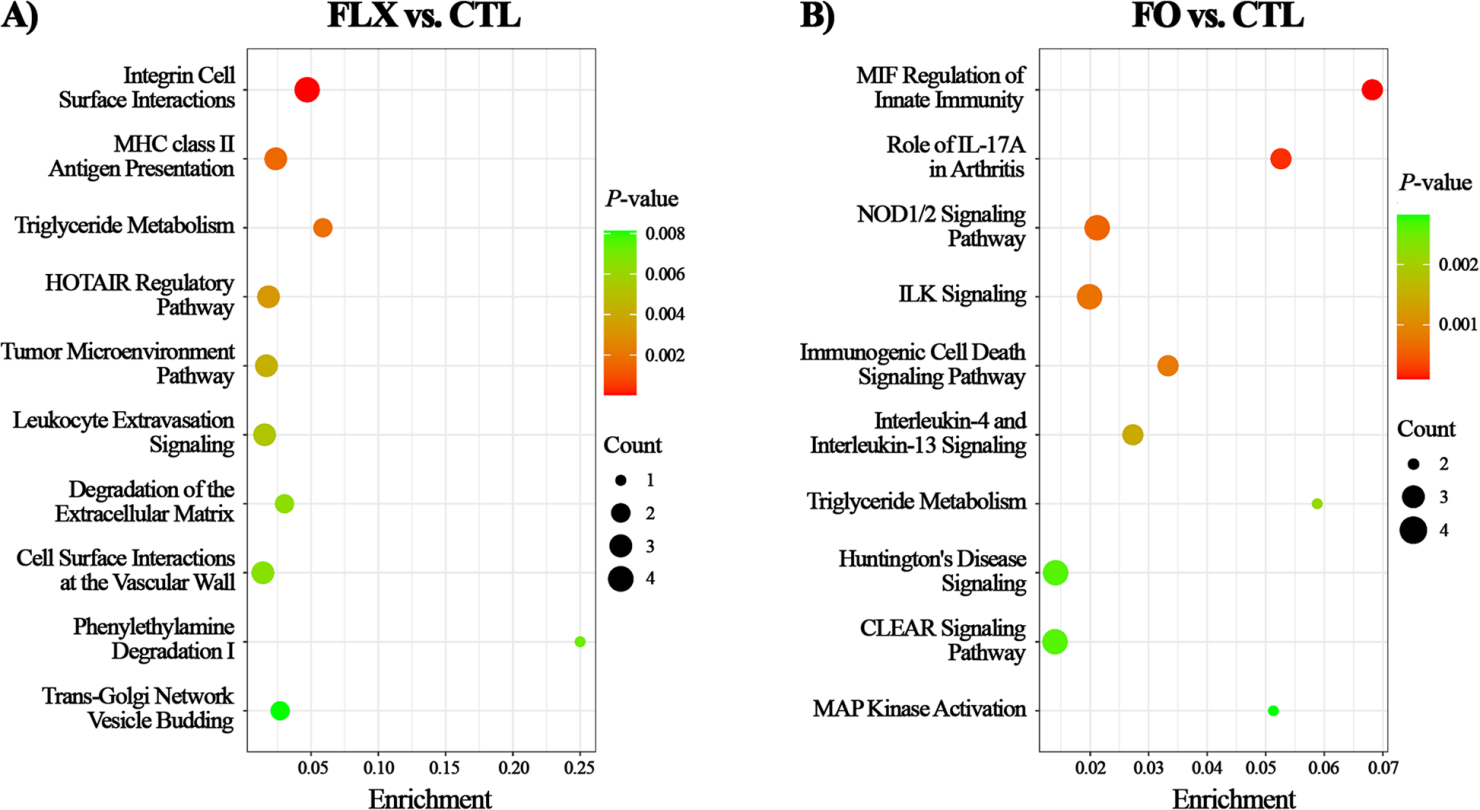
Top canonical pathway analysis according to differential proteome in placenta FLX or FO vs. CTL. Proteome analysis in placenta of dairy cows supplemented pre-partum with (i) CTL – encapsulated saturated fat, (ii) FLX – encapsulated flaxseed oil providing ALA, or (iii) FO – encapsulated fish oil providing EPA and DHA. (**A**) FLX vs. CTL and (**B**) FO vs. CTL. Enrichment (*X*-axis) is calculated by dividing the number of DAPs (|FC| ≥ 1.5) assigned to a particular pathway by the total number of molecules within that pathway. *P*-value (threshold of ≤ 0.05) is depicted by color scale. Plots were generated using SRplot [41]

The top canonical pathways enriched in FLX vs. CTL placenta (Fig. 3A) were integrin cell surface interactions, major histocompatibility complex (MHC) class II antigen presentation, triglyceride metabolism, homeobox transcript antisense intergenic RNA (HOTAIR) regulatory pathway, and leukocyte extravasation signaling. Most pathways were mainly based on the differential abundance of FABP3, FABP7, CD44, and SPP1.

In comparing FO to CTL (Fig. 3B), the top canonical pathways enriched were macrophage migration inhibitory factor (MIF) regulation of innate immunity, NOD1/2 signaling pathway, triglyceride metabolism, and mitogen-activated protein (MAP) kinase activation. Most pathways were based mainly on the differential abundance of FABP3, FABP7, PTGS2, and Nitric oxide synthase (NOS2). In comparing FO to FLX (Additional file 1: Fig. S3), the top canonical pathways annotated were RHO GTPases that activate protein kinases N (PKNs), triglyceride metabolism, triacylglycerol biosynthesis, and neutrophil degranulation. Most pathways were based mainly on the differential abundance of FABP5, phosphatidate phosphatase (LPIN2), and lysophosphatidylcholine acyltransferase 1 (LPCAT1).

#### Biological functions and networks

**Fig. 4:**
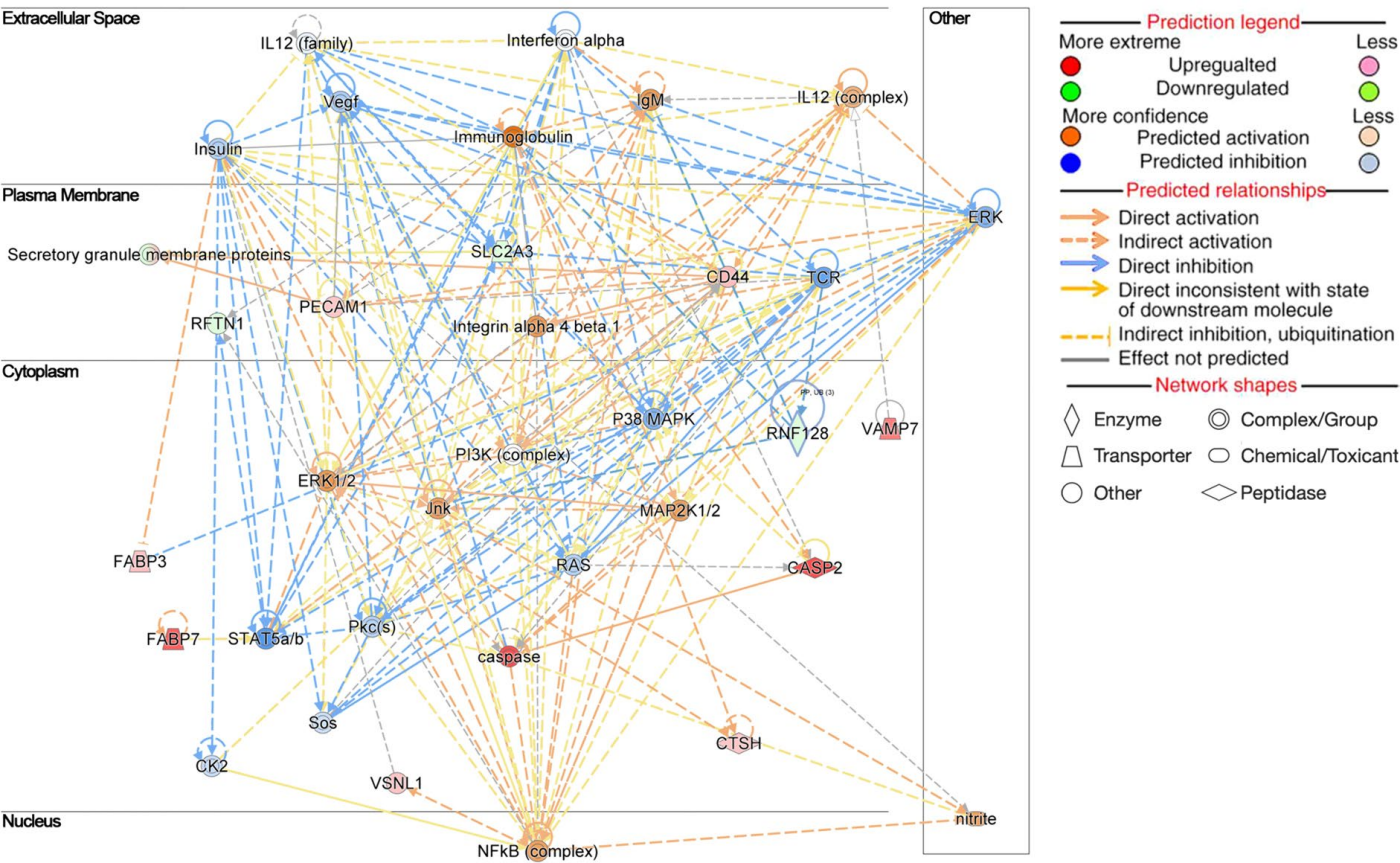
Selected top scoring biological networks obtained for FLX vs. CTL using IPA analysis. Network ‘Lipid Metabolism, Molecular transport, and Protein synthesis’ includes the DAPs: *CASP2* Caspase 2, *CD44* Antigen CD44, *FABP3* Heart-type fatty acid binding protein, *FABP7* Brain-type fatty acid binding protein, brain, *NFkB-complex* NFKB2 protein, and *P38 MAPK* p38 mitogen-activated protein kinase. The image was generated using www.qiagen.com/ingenuity

Among the top biological functions enriched in FLX vs. CTL (Table 2), those related to the FA metabolism (*P* = 0.0003), the concentration of lipid (*P* = 0.002), the synthesis of ROS (*P* = 0.02), the inflammation of organs (*P* = 0.000001), the inflammation of the absolute anatomical region (*P* = 0.0005), and the inflammation of the body cavity (*P* = 0.002) were most likely activated. In contrast, the biological functions related to the concentration of triacylglycerol (*P* = 0.004) were most likely inhibited. One of the main networks affected in FLX vs. CTL according to the DAP was lipid metabolism, molecular transport, and protein syntheses (Fig. 4). Some of the components related to the lipid metabolism network were CASP2, CD44, FABP3, FABP7, MAP2K1 protein (MAP2K1/2), NFKB2 protein (NFkB-complex), p38 mitogen-activated protein kinases (P38 MAPK), and platelet endothelial cell adhesion molecule (PECAM1).

**Table 2:** Top biological functions identified by IPA software based on differentially abundant proteins

Treatment^1^/Functions annotation	*P*-value (z-score^2^)	Proteins associated with function
**CTL vs. FLX**		
Concentration of lipid	0.002 (0.644)	CASP2, FABP3, FKBP4, GBA1, ICMT, NQO1, SAR1B, SLC6A11, SPP1
Concentration of triacylglycerol	0.004 (–0.046)	CASP2, FKBP4, NQO1, SAR1B, SPP1
Fatty acid metabolism	0.0003 (1.318)	CYP2S1, FABP3, FABP7, GBA1, NQO1, SAR1B, SLC6A11, VAMP7
Synthesis of ROS	0.02 (1.295)	ATG16L1, CD44, NQO1, PECAM1, SPP1
Inflammation of organ	0.00001 (0.364)	ATG16L1, CA2, CASP2, CD44, CTSH, FKBP4, GBA1, GFAP, NQO1, PECAM1, RFTN1, RNF128, S100A4, SAR1B, SPP1, TKT
Inflammation of absolute anatomical region	0.0005 (0.102)	ATG16L1, CA2, CASP2, CD44, GBA1, GFAP, NQO1, PECAM1, RFTN1, RNF128, S100A4, SAR1B, SLC15A1, SPP1, TKT
Inflammation of body cavity	0.002 (0.78)	ATG16L1, CASP2, CD44, NQO1, PECAM1, RNF128, S100A4, SAR1B, SPP1, TKT
**FO vs. CTL**		
Concentration of fatty acid	0.02 (–1.49)	CLN3, FABP3, NOS2, PTGS2
Fatty acid metabolism	0.01 (0.44)	CLN3, FABP3, FABP7, MAPK9, NOS2, PTGS2
**FO vs. FLX**		
Synthesis of lipid	0.03 (–1.205)	AKR1B1, FABP5, LPCAT1, LPIN2, SGPP1, YWHAG
Concentration of lipid	0.006 (0.277)	AKR1B1, FABP5, FUCA1, LIMA1, LPCAT1, RBP4, SGPP1
Synthesis of ROS	0.01 (–0.323)	AHSP, AKR1B1, CFH, CTTN, GPX3, RBP4
Production of ROS	0.04 (–0.152)	AHSP, AKR1B1, CTTN, RBP4
Inflammation of absolute anatomical region	0.04 (–0.651)	CA2, CAST, CFH, CTTN, DYM, EEF1E1, GPX3, ITFG1, NT5E, RBP4, SGPP1

^1^Dairy cows at d 257 of pregnancy were divided into three nutritional groups supplemented with (i) CTL – encapsulated saturated fat, (ii) FLX – encapsulated flaxseed oil providing ALA, or (iii) FO – encapsulated fish oil providing EPA and DHA.

^2^z-score represents the inferred activation (z-score > 0) or inhibition (z‐score < 0)

*AHSP* Alpha-hemoglobin-stabilizing protein, *AKR1B1* Aldo-keto reductase family 1 member B1, *ATG16L1* Autophagy related 16 like 1, *CA2* Carbonic anhydrase 2, *CASP2* Caspase 2, *CD44* CD44 antigen, *CFH* Complement factor H, *CLN3* Battenin, *CTSH* Cathepsin H, *CTTN* Cortactin, *CYP2S1* Cytochrome P450, *DYM* Dymeclin, *FABP3* Heart-type fatty acid-binding protein, *FABP5* Epidermal-type fatty acid-binding protein, epidermal, *FABP7* Brain-type fatty acid-binding protein, brain, *FKBP4* Peptidylprolyl isomerase, *GBA1* Glucosylceramidase Beta 1, *GFAP* Glial fibrillary acidic protein,*GPX3* Glutathione peroxidase 3, *ICMT* Protein-S-isoprenylcysteine O-methyltransferase, *LPCAT1* Lysophosphatidylcholine acyltransferase 1, *LPIN2* phosphatidate phosphatase, *MAPK9* Stress-activated protein kinase JNK, *NQO1* NAD(P)H quinone dehydrogenase 1, *NOS2* Nitric oxide synthase, *PECAM1* Platelet endothelial cell adhesion molecule, *PTGS2* Prostaglandin-endoperoxide synthase 2, *RBP4* Retinol-binding protein 4, *RFTN1* Raftlin, lipid raft linker 1, *RNF128* Ring finger protein 128, *S100A4* S100 calcium-binding protein A4, *SAR1B* GTP-binding protein SAR1b, *SGPP1* Sphingosine-1-phosphate phosphatase 1, *SLC15A1* Solute carrier family 15 member 1, *SLC6A11* Transporter, *SPP1* Secreted phosphoprotein 1, *TKT* Transketolase, *VAMP7* Vesicle-associated membrane protein 7, *YWHAG* Tyrosine 3-monooxygenase. *ROS* Reactive oxygen species

Regarding FO vs. CTL (Table 2), the biological function of proteins related to FA metabolism (*P* = 0.01) was most likely activated, whereas functions related to the concentration of FAs (*P* = 0.02) were most likely inhibited. The top enriched molecular network regarding FO vs. CTL was cardiovascular system development and function, organ morphology, and organismal development (Additional file 1: Fig. S4A). Some of the components related to this network were caspase, CD59 molecule (CD59), FABP3, NOS2 and PTGS2.

Regarding FO vs. FLX (Table 2), the biological function affecting the concentration of lipid (*P* = 0.02) was most likely activated, whereas functions related to the synthesis of lipid (*P* = 0.03), production of ROS (*P* = 0.04), synthesis of ROS (*P* = 0.01), and inflammation of absolute anatomical region (*P* = 0.04) were most likely weakly inhibited. In comparing FO to FLX, one of the top networks affected was the cellular development, dermatological diseases and conditions, organismal injuries and abnormalities network (Additional file 1: Fig. S4B). The main components of this network are FABP5, NFkB complex, P38 MAPK, and complement factor H (CFH).

### Docking studies of endocannabinoids with bovine FABPs

**Fig. 5:**
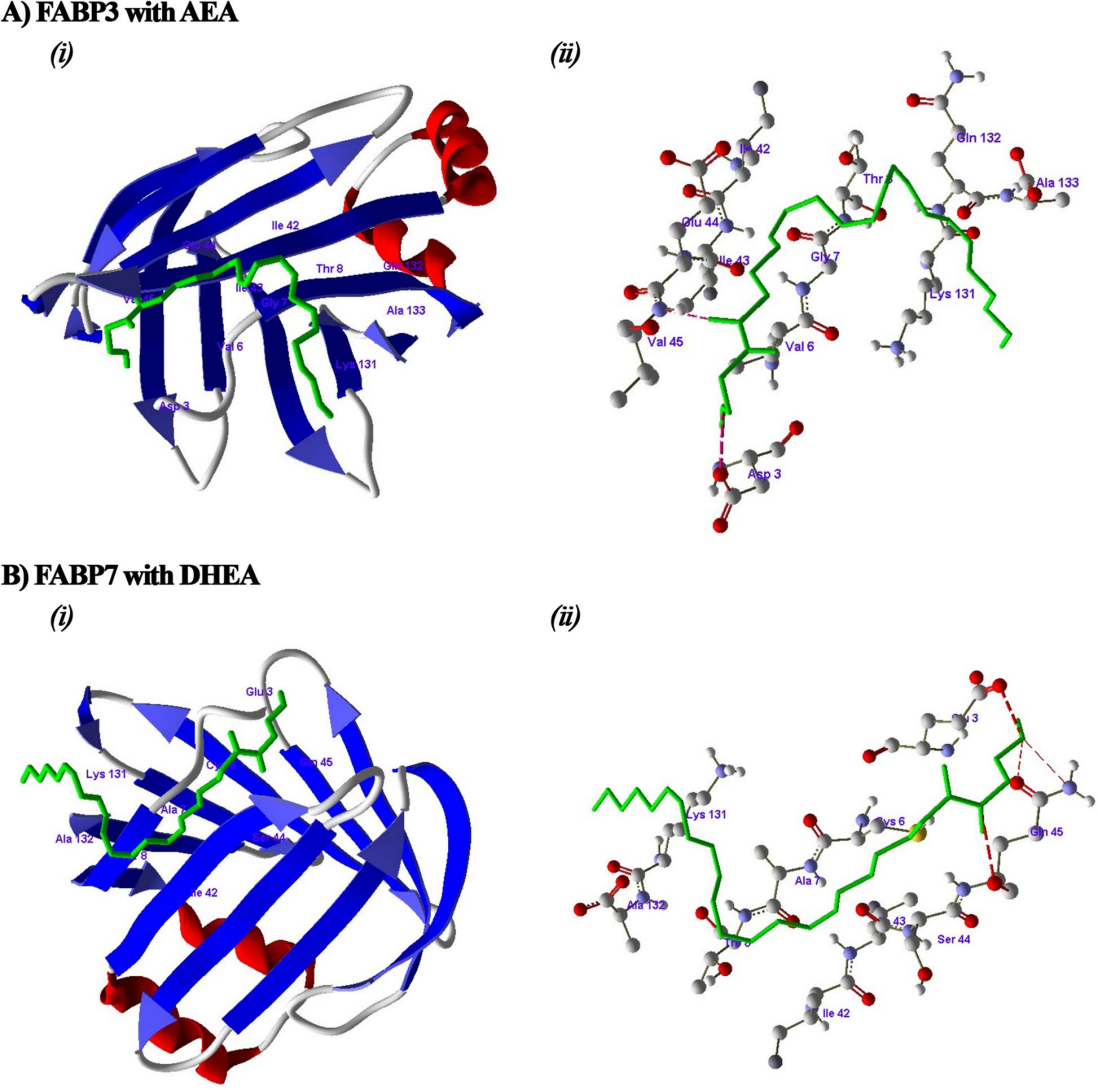
*In silico* binding studies of the endocannabinoids with FABPs proteins from *Bos taurus*. (**A**) Docking studies of AEA with FABP3 and amino acids involved in hydrogen bonding with the AEA. (**B**) Docking studies of DHEA with FABP7 and amino acids involved in hydrogen bonding with the DHEA; hydrogen bonds were denoted by red dotted lines

*In-silico* docking studies performed on bovine FABPs confirmed that FABP3 binds the n-6 series endocannabinoids AEA (4.16 kcal/mol, Fig. 5A) and 2-AG (6.35 kcal/mol, Additional file 3: Fig. S4) with good fitness scores. 2-AG and AEA also bind to FABP5 (5.90 kcal/mol and 3.55 kcal/mol, respectively) with good fitness scores (Additional file 3: Fig. S5). On the other hand, 2-AG and AEA had low fitness scores when binding to FABP7 (0.06 kcal/mol and 0.15 kcal/mol, respectively; Additional file 3: Fig. S6); however, as shown in Fig. 5B, FABP7 had a good binding score with the n-3 series endocannabinoid DHEA (10.43 kcal/mol).

### Effects of maternal n-3 FA supplementation on FA composition in the placenta and plasma of neonate calves and on the inflammatory markers in neonate calves

First, we assessed the effects of maternal n-3 FA supplementation on the FA profile in the placenta tissues and the plasma of neonate calves. As shown in Table 3, in the placenta tissues, the average percentages of the n-3 FAs and the n-6/n-3 FA ratio remained similar across the treatments. On the other hand, in neonate calves, the plasma FA profile was affected by the maternal supplementation of the n-3 FAs; FO calves had a higher average percentage of total n-3 FAs (*P* = 0.02) compared with CTL (Table 4). Furthermore, a larger number of FO calves displayed increased percentages of plasma C20:5n-3 (EPA, *P* = 0.001), C22:6n-3 (DHA, *P* = 0.003), and their intermediate C22:5n-3 (DPA, *P* = 0.003), compared with both the CTL and FLX calves (Table 4).

**Table 3:** Placenta fatty acid profile from cows supplemented with saturated fat, flaxseed oil, or fish oil

FA, %	Treatment^1^			SEM	* P*-value
	CTL	FLX	FO		
C14:0^2^	1.40	1.22	1.37	0.06	0.15
C16:0^2^	27.43	25.31	26.84	1.53	0.62
C16:1^2^	0.84^b^	2.71^ab^	3.83^a^	0.58	0.03
C16:2^2^	6.68^a^	1.53^b^	0.85^b^	1.02	0.0002
C16:3^2^	0.08	0.14	0.04	0.05	0.52
C18:0^2^	9.78	14.89	12.72	2.80	0.48
C18:1n-9^2^	37.10	33.53	35.39	2.71	0.67
C18:1n-7^2^	0.26	0.22	0.16	0.04	0.33
C18:2n-6^2^	7.25	8.75	8.96	1.43	0.67
C18:3n-3 (ALA)^2^	4.66	5.52	4.75	0.40	0.32
C20:4n-6^2^	4.22	4.28	3.17	0.91	0.64
C20:5n-3 (EPA)^2^	0.79	0.87	0.57	0.13	0.31
C22:5n-3^2^	0.03	0.04	0.05	0.01	0.45
C22:6n-3 (DHA)^2^	1.16	0.98	1.30	0.17	0.47
SFA	38.62	41.42	40.94	1.53	0.43
MUFA	38.20	36.46	39.37	2.29	0.68
PUFA	23.18	22.12	19.69	1.19	0.18
Total n-3 FAs	6.64	7.41	6.66	0.60	0.61
Total n-6 FAs	11.48	13.03	12.13	1.87	0.84
n-6:n-3 FAs ratio	1.71	1.76	1.94	0.36	0.89

^1^Dairy cows at d 257 of pregnancy were divided into three nutritional groups supplemented with (i) CTL – encapsulated saturated fat, (ii) FLX – encapsulated flaxseed oil providing ALA, or (iii) FO – encapsulated fish oil providing EPA and DHA

^2^C14:0 to C22:6n-3: % of total FAs identified

*ALA* Alpha-linolenic acid, *DHA* Docosahexaenoic acid, *EPA* Eicosapentaenoic acid, *FAs* Fatty acids, *SFA* Saturated FA, *MUFA* Monounsaturated FA, *PUFA* Polyunsaturated FA

^a,b^Values with different superscript letters in a row are significantly different at *P* ≤ 0.05

**Table 4:** Plasma fatty acid profile in neonatal calves born to cows supplemented with saturated fat, flaxseed oil, or fish oil

FA, %	Treatment^1^			SEM	* P*-value
	CTL	FLX	FO		
C14:0^2^	5.80	4.26	5.43	0.58	0.17
C16:0^2^	34.76	31.34	32.77	1.35	0.25
C16:1^2^	1.14	1.15	0.86	0.10	0.096
C16:2^2^	4.19	4.22	4.00	0.23	0.78
C16:3^2^	0.51	0.62	0.54	0.07	0.49
C18:0^2^	10.42	11.10	10.34	0.43	0.41
C18:1n-9^2^	22.83	24.53	24.16	1.02	0.47
C18:1n-7^2^	2.22	2.39	2.12	0.17	0.52
C18:2n-6^2^	14.19	16.18	15.10	1.65	0.69
C18:3n-3 (ALA)^2^	1.12	1.24	1.16	0.07	0.46
C20:4n-6^2^	2.66	3.18	2.86	0.22	0.29
C20:5n-3 (EPA)^2^	0.02^b^	0.01^b^	0.13^a^	0.02	0.002
C22:5n-3^2^	0.02^b^	0.02^b^	0.20^a^	0.03	0.001
C22:6n-3 (DHA)^2^	0.04^b^	0.01^b^	0.17^a^	0.03	0.002
SFA	49.35	46.70	48.53	1.28	0.34
MUFA	26.33	28.07	27.14	1.15	0.56
PUFA	22.67	25.22	24.32	1.90	0.62
Total n-3 FAs	1.19^b^	1.29^ab^	1.59^a^	0.11	0.05
Total n-6 FAs	16.85	19.19	17.96	1.82	0.66
n-6:n-3 FAs ratio	14.20	14.88	11.43	1.01	0.06

^1^Dairy cows at d 257 of pregnancy were divided into three nutritional groups supplemented with (i) CTL – encapsulated saturated fat, (ii) FLX – encapsulated flaxseed oil providing ALA, or (iii) FO – encapsulated fish oil providing EPA and DHA

^2^C14:0 to C22:6n-3: % of total FAs identified

*ALA* Alpha-linolenic acid, *DHA* Docosahexaenoic acid, *EPA* Eicosapentaenoic acid, *FAs* Fatty acids, *SFA* Saturated FA, *MUFA* Monounsaturated FA, *PUFA* Polyunsaturated FA

^a,b^Values with different superscript letters in a row are significantly different at *P* ≤ 0.05

Next, we evaluated the potential impact of maternal dietary n-3 FAs supplementation on the inflammatory status of neonatal calves by quantifying the plasma concentrations of several inflammatory markers on the day of calving before colostrum offering. A significant reduction of 14% and 22% in the average plasma concentrations of IL-6 was observed in the FLX and FO calves, respectively, compared with the CTL calves (*P* = 0.001; Table 5). On the other hand, there was no significant difference in IL-2 concentrations in both n-3 FAs treatments compared to CTL (Table 5); however, it was 22.8% higher in FLX than in the FO calves (*P* = 0.02). No differences were observed regarding the average concentrations of haptoglobin among the treatments (Table 5).

**Table 5:** Average concentrations of inflammatory markers in plasma of neonatal calves before colostrum intake

Variable	Treatment^1^			SEM	* P*-value
	CTL	FLX	FO		
IL-2, pg/mL	372.7^ab^	427.6^a^	329.9^b^	22.76	0.020
IL-6, pg/mL	209.5^a^	179.6^b^	162.6^b^	7.66	0.001
Haptoglobin, ng/mL	27.0	24.5	26.0	6.89	0.960

^1^Blood samples were collected immediately after calving from calves born to dairy cows fed from d 257 of pregnancy with (i) CTL – encapsulated saturated fat, (ii) FLX – encapsulated flaxseed oil providing ALA, or (iii) FO – encapsulated fish oil providing EPA and DHA

*IL-2* Interleukin 2, *IL-6* Interleukin 6

^a,b^Values with different superscript letters in a row are significantly different at *P* ≤ 0.05

## Discussion

Dietary n-3 FAs positively affect the physiological and reproductive properties in dairy cows [45]. In this work we aimed to investigate how maternal supplementation with different sources of n-3 FAs affects the genes and proteins related to the main physiological pathways in the placenta of dairy cows. We hypothesized that maternal n-3 FA supplementation would modulate lipid metabolism by improving the transfer of n-3 FAs through the placenta to the fetus, down-regulate ECS components, and have anti-inflammatory and anti-oxidative effects that may be beneficial for placental function and for the inflammatory response in neonates. Indeed, our proteomic approach, combined with gene expression data, showed that providing late-pregnant cows with FLX or FO, both sources of n-3 FAs, affects the bovine placenta via alterations in the expression patterns of proteins and genes related to these processes. Our findings of increased levels of inflammatory and ECS components in the FO placenta were unexpected, and will be discussed next.

### Maternal n-3 FA supplementation affects the placental lipid metabolism

The fetus is dependent on the maternal supply of LCPUFAs; thus, the maternal FA composition is crucial for fetal growth and development, especially during late pregnancy, when DHA is particularly important for fetal brain development [46]. Placental cells are known to transfer the FAs selectively, with a preference for LCPUFAs, such as EPA and DHA. The selective uptake may involve intracellular metabolic channeling and a selective supply to the fetal circulation [2, 47, 48]. Several protein families were implicated in directional FA transport in the placenta: the membranal FATPs, FA translocase (FAT/CD36), and the cytoplasmic FABPs [2, 48, 49]. The uptake and accumulation of FAs are regulated by FABPs; FABP3, FABP4, FABP5, and FABP7 are expressed in cells with high FA requirements, such as the placenta [48, 50, 51]. In this study, we found that several FABPs were upregulated in FLX and/or the FO placenta; the abundances of FABP3 and FABP7 increased in both n-3 FAs groups, and these increases were related to the enriched pathway of triglyceride metabolism, whereas FABP4 gene expression was augmented by FO supplementation. Furthermore, FABP5 was higher in FO than in FLX placenta. FABPs have differential affinities to specific FAs: FABP5 binds to both DHA and AA, whereas FABP7 binds specifically to DHA [47, 52–54], and both FABP4 and FABP5 specifically increase the uptake of DHA [55–58]. Furthermore, although FABP3 was shown to bind preferentially to AA (n-6 FA) [54], it regulates both n-3 and n-6 FA transport in mouse trophoblasts [59]. Studies in bovine ovarian granulosa [60] and human placental cells [61] showed that the expression of FABP3, which may regulate the accumulation of triglycerides and the capacity of lipid transfer, can be influenced by the type of LCPUFA and the maternal health condition. In the present study, the plasma FA profile of neonate FO calves showed an increase in total n-3 FAs compared with CTL. We have previously demonstrated that the maternal plasma of FO cows was enriched with DHA and EPA [30]. Taken together, along with the increase in specific placental FABPs, we suggest that the increased maternal n-3 FAs may lead to increased FABPs in the placenta, which favors the trafficking of n-3 FAs to the fetus. The apparent lack of the effect of the dietary FA supplementation on the FA profile in placental cotyledons may stem from a highly efficient maternal-fetal transfer and/or rapid utilization of the FAs for placental functions.

Our proteomic data showed an increased abundance of CASP2 in the FLX placenta, compared with CTL. CASP2 is a protein in the apoptotic cascade, which was recently identified as a positive regulator of cholesterol and triacylglycerol homeostasis in human cells and mice [62, 63]. According to our bioinformatic analysis, CASP2 is involved in lipid concentration and triglyceride metabolism; it was demonstrated that the human CASP2 gene contains multiple sites that can be recognized by transcriptional regulators of the sterol regulatory element-binding protein (SREBP) family [62], which is involved in regulating pathways for cholesterol, triacylglycerol, and phospholipid synthesis [64]. In the placenta, the FAs are esterified into triglycerides and stored in droplets within the cells, after which they are transferred to the developing fetus [65]; thus, variations in the abundance of CASP2 may lead to modification of the cellular lipid levels, and specifically triacylglycerol [62, 63]. Therefore, our results suggest that maternal n-3 FA supplementation may modulate the metabolic pathway of triacylglycerol/lipid in the placenta by affecting the abundance of CASP2 protein.

Taken together, our results suggest that FO had a greater effect on the transfer of n-3 LCPUFAs to the fetus than did FLX. This transfer may be specifically mediated by modulating the FABPs in the placenta.

### Maternal n-3 FA supplementation modulates ECS components in the placenta

Changing the n-3 to n-6 ratio in the diet is a well-known strategy to modulate the ECS [66]. We previously reported that in dairy cows, peripartum n-3 FA supplementation reduces the abundance of certain ECS components in the adipose tissue, liver, and white blood cells [24, 31]. However, in this study, in the placental tissue, FLX supplementation did not affect the gene expression of most of the examined ECS components, except for the *FAAH* gene. Intriguingly, the placenta from FO cows exhibited a higher gene expression of the primary membranal ECS receptors (*CNR1* and *CNR2*), and a tendency toward increased gene expression of a secondary ECS nuclear receptor *PPARG*. Both the CNR1 and CNR2 receptors have important functions in reproductive organs, including the placenta [67, 68], since activation of the ECS receptors can affect both pregnancy progression and labor [29, 67] by controlling the action of endocannabinoids, cytokine release, and by modulating the mitochondrial activity [69, 70]. Within the cells, n-3 FA DHA and EPA can be converted to the endocannabinoids DHEA and eicosapentaenoyl ethanolamide (EPEA), which have a chemical structure similar to the endocannabinoid AEA; it was reported that DHEA and EPEA exhibit binding affinity and agonist activity on both CNR1 and CNR2 [71, 72]. Thus, the effects of FO on the ECS can be mediated by these n-3 FA-derived endocannabinoids. However, in the present study we did not measure the levels of endocannabinoids in placenta; therefore, further studies are required to investigate this possibility.

Interestingly, FABP3, FABP5, and FAB7 are also involved in the uptake, intracellular transport, and hydrolysis of endocannabinoids [51, 55, 73–76]. Since our data showed increased abundances of several FABPs in n-3 FA supplemented placentas, together with modulations in the ECS components, we investigated, for the first time in bovine, the *in-silico* binding of these *Bos taurus* FABPs with endocannabinoids. We found that FABP3 and FABP5 showed good fitness scores in the docking with both 2-AG and AEA, whereas bovine FABP7 had a good fitness score with DHEA. These findings may indicate an association between lipid metabolism and ECS within the bovine placenta. Furthermore, we observed a lower abundance of CNRIP1 protein in FO compared with FLX. CNRIP1 is involved in modulating part of the CNR1, thereby regulating cell signaling initiated by modulation of CNR1 [77]. However, the function of CNRIP1 in placental cells is not yet fully understood. To the best of our knowledge, this is the first study to identify CNRIP1 in the synepitheliochorial placenta, and there is a suggested link between n-3 FA supplementation and this ECS component.

Modulating the ECS can also affect inflammatory processes. In myoblast cell cultures, EPEA and DHEA decreased the gene expression of the inflammatory marker *IL-6* [78]. However, in our study, the upregulation of the CNR in FO placenta coincided with a tendency to increase *IL-6* expression. On the other hand, both FO and FLX placentas had reduced gene expression of *FAAH* compared with CTL. FAAH inhibition is considered to have anti-inflammatory effects [79, 80]. Taken together, we propose that maternal FO supplementation can stimulate placental ECS, which may be associated with activating the inflammatory and lipid metabolism pathways.

### Maternal n-3 FA supplementation affects inflammation in the placenta and in calves

Within the placenta, pro-inflammatory cytokines are produced during parturition, most likely contributing to uterine contractions and the expulsion of the fetus [81, 82]. Note that we specifically sampled the cotyledons of the expelled placentas, namely, the maternal-fetal interface tissues post-detachment. When we examined the expression of inflammatory genes in the placental cotyledons, we found an increase in the expression of anti-inflammatory *IL-10* and a tendency toward a higher expression of pro-inflammatory *IL-6* in FO compared with CTL. In addition, an increase in the protein abundance of CD44 and SPP1, both associated with the activation of immune cells during inflammation [83], was found in FLX compared to CTL, and SPP1 tended to be increased in FO compared with CTL. Moreover, our analysis revealed an enrichment of the MHC class II antigen presentation and the leukocyte extravasation signaling pathways, as well as in the connection to inflammatory molecules, such as p38 MAPK and NFkB in the network, which are pro-inflammatory, in FLX vs. CTL [84]. In addition, in FO vs. CTL we found an enrichment in the MIF regulation of innate immunity, NOD1/2 signaling, and the MAP kinase activation pathways, which are associated with activation and control of the inflammatory process [85–87]. These findings indicate that maternal supplementation of n-3 FAs mostly upregulates the inflammatory process in the expelled placenta, which contradicts the well-described anti-inflammatory effects of n-3 FAs in other tissues. However, our analyses were conducted specifically on placental cotyledons, revealing a specific localized pro-inflammatory response. This observation aligns with the findings of Peng et al. [23], who demonstrated that EPA supplementation can induce a distinctive local pro-inflammatory effect in placenta from mice. However, in contrast to the putative upregulation of the inflammatory processes in the placenta, the proteomic data in FO vs. CTL showed a decreased abundance of PTGS2, encoding the enzyme cyclooxygenase-2, which regulates the synthesis of pro-inflammatory prostaglandins [88, 89]. The lower abundance of PTGS2 may indicate the anti-inflammatory effects of FO, which is in line with the increased gene expression of *IL-10*, thus overall, suggesting that FO has both pro- and anti-inflammatory effects on the bovine placenta.

In the present study we also investigated the effects of maternal n-3 FA supplementation on inflammatory markers in the neonate calves. We found that both FLX and FO reduced the plasma concentrations of IL-6 in neonates, which is in accordance with the well-described anti-inflammatory effects of n-3 FAs. Although the placental synepteliochorial does not allow the transfer of large molecules, such as immunoglobulins and lipid-insoluble molecules from the maternal blood flow to the fetus [12, 90], based on our results, it is possible that during late pregnancy, the maternal diet can influence the inflammatory response of neonates before the ingestion of colostrum, by facilitating placental transfer of n-3 FAs that modulate the release of cytokines, thus, highlighting the role of the placenta in the immune status of neonates during their first hours of life.

### Maternal n-3 FA supplementation affects the placental oxidative stress response

The placental cells exhibit a high level of mitochondrial activity, which leads to the production of ROS [17] and increased oxidative stress [91]. Our proteomic data indicated that ROS synthesis was altered significantly in FLX placenta compared with CTL. In addition, we identified the up-regulation of *CAT* expression in FO placentas, whereas the expressions of *GPX3* and *SOD1* were similar across the treatments. In rats, n-3 FA supplementation increases the oxidative stress response in placental tissue, along with a positive correlation with the gestational quality [92]. Dietary n-3 PUFAs, and specifically DHA, were shown to act as activators of the Nrf2 antioxidant pathway [93–96]. Nrf2 is a transcriptional activator that regulates the expression of antioxidant genes including *SOD1*, *NQO1*, *CAT*, and *GPX3* [97]. Indeed, we found that the abundance of NQO1 increased in the placental cotyledon cells of the FLX group. The NQO1 is a cytoplasmic protein that plays a key role in protecting against oxidative stress by stabilizing various proteins and preventing the reduction of a single electron that leads to the production of ROS [8]. We found that in FLX vs. CTL, NQO1 was most likely activated in biological functions related to ROS synthesis. Interestingly, although the analysis of the proteomic data did not reveal a significant impact on the ROS-related functions in the FO placenta, we did find an increased gene expression of the antioxidant enzyme *CAT* in FO compared with CTL. In a study with diabetic rats, supplementation with FO increased the gene expression of *CAT*, which is in agreement with our findings [98]. In the present study, there was a decrease in the gene expression of the ECS enzyme *FAAH* in both FO and FLX, compared with CTL. FAAH degrades AEA into ethanolamine and AA. AA, besides constituting a high percentage of the membrane phospholipids in cells, also serves as a precursor for the synthesis of prostaglandins-ethanolamids or ‘prostamides’, such as prostamide-E2 (PME2) [5, 89]. PME2 exhibits proapoptotic action and can lead to the production of ROS [68, 89]; therefore, reduction of FAAH may lessen oxidative stress.

In comparing the FO and FLX treatments, two functions associated with the production and synthesis of ROS were most likely inhibited: One of the proteins assigned to this function was GPX3, a member of the enzyme antioxidant family whose main biological role is to minimize oxidative damage by catalyzing the removal of lipid peroxides, which are products of ROS activity [99]. Indeed, GPX3 abundance was upregulated in FO compared with FLX. In sheep, Garrel et al. [100] suggested that GPX may be the major enzyme in defending against ROS in the fetus-placental unit. Overall, the increase in the oxidative stress response in the n-3 FA supplemented placental tissues is important to preserve gestational function [92]. An important consideration in interpreting our results is the specific highly- specialized tissue examined and the timing of the sample collection, which displays a physiological increase in the oxidative processes. Based on our results indicating that several oxidative stress components at the transcriptional and post-transcriptional levels were stimulated both in FLX and in FO, we suggest that maternal supplementation of n-3 FAs can have antioxidant effects on the bovine placenta.

## Conclusions

Maternal n-3 FA from FLX and FO differentially affected the bovine placenta; both enhanced lipid metabolism and modulated oxidative stress; however, FO increased some transcriptional ECS components, possibly related to the increased FABPs. Maternal FO induced a unique balance of pro- and anti-inflammatory components in the placenta. Both n-3 FA supplementations altered the inflammatory markers in neonatal blood. Taken together, different sources of n-3 FA during late pregnancy affected placental immune and metabolic processes, which may affect the neonatal immune system.

### Supplementary Information

**Additional file 1: Fig. S1. **Schematic representation of the experimental procedures and analyses performed in this study.** Fig. S2. **Functional categorization of DAPs in IPA. **Fig. S3.** Top canonical pathways according to the differential proteome analysis in placenta FO vs. FLX.** Fig. S4. **Selected networks based on IPA analysis of in FO vs. CTL and FO vs. FLX.

**Additional file 2. **Proteomic analysis results.

**Additional file 3. ***In-silico* docking studies of bovine FABPs with selected endocannabinoids. Detailed description of the methods and results of the analysis.

## Data Availability

The proteomic dataset generated and/or analyzed during the current study is available in the Supplementary files.

## Abbreviations

2-AG: 2-arachidonoylglycerol
AA: Arachidonic acid
ACTB: β-Actin
AEA: Anandamide
AGC: Automatic gain control
ALA: Alpha-linolenic acid
CASP2: Caspase 2
CAT: Catalase
CD44: CD44 antigen
CD59: CD59 molecule
CFH: Complement factor H
CNR1: Cannabinoid receptor 1
CNR2: Cannabinoid receptor 2
CNRIP1: CNR1-cannabinoid receptor interacting protein 1
CTL: Control group
DAP: Differently abundant protein
DHA: Docosahexaenoic acid
DHEA: Docosahexaenoyl ethanolamide
DPA: Docosapentanoic acid
ECS: Endocannabinoid system
ELISA: Enzyme-linked immunosorbent assays
EPA: Eicosapentaenoic acid
EPEA: Eicosapentaenoyl ethanolamide
FA: Fatty acid
FAAH: Fatty acid amide hydrolase
FABP3: Heart-type fatty acid binding protein
FABP4: Fatty acid binding protein 4
FABP5: Epidermal-type fatty acid-binding protein
FABP7: Brain-type fatty acid binding protein
FABP: Fatty acid binding protein
FASN: Fatty acid synthase
FAT/CD36: Fatty acid translocase
FATPs: Fatty acid transport proteins
FC: Fold change
FLX: Flaxseed oil
FO: Fish oil
GAPDH: Glyceraldehyde-3-phosphate dehydrogenase
GLM: Generalized linear model
GPX3: Glutathione peroxidase 3
HP: Haptoglobin
HOTAIR: Homeobox transcript antisense intergenic RNA
IL-1b: Interleukin 1b
IL-2: Interleukin 2
IL-6: Interleukin 6
IL6-R: Interleukin-6 receptor
IL-10: Interleukin 10
IPA: Ingenuity® pathway analysis
KOH: Potassium hydroxide
LC/MS: Liquid chromatography tandem mass spectrometry
LCPUFA: Long-chain polyunsaturated fatty acids
LPCAT1: Lysophosphatidylcholine acyltransferase 1
LPIN2: Phosphatidate phosphatase
MAP: Mitogen-activated protein
MAP2K1/2: MAP2K1 protein
MGLL: Monoglyceride lipase
MIF: Macrophage migration inhibitory factor
MHC: Major histocompatibility complex
n-3 FA: Omega-3 fatty acid
n-6 FA: Omega-6 fatty acid
NAPEPLD: N-acyl phosphatidylethanolamine phospholipase D
NCE: Normalized collision energy
NFkB: Nuclear factor kappa B
NFkB-complex: NFKB2 protein
NanoESI: Nano-electrospray ionization mass spectrometry
NOS2: Nitric oxide synthase
NQO1: NAD(P)H quinone dehydrogenase 1
P38 MAPK: p38 mitogen-activated protein kinases
PCA: Principal component analysis
PCR: Polymerase chain reaction
PECAM1: Platelet endothelial cell adhesion molecule
PKNs: Protein kinases N
PME2: Prostamide-E2
PMSF: Phenylmethylsulfonyl fluoride
PPARG: Proliferator-activated- receptor gamma
PPM: Parts per million
PSM: Peptide-spectrum match
PTGS2: Prostaglandin–endoperoxide synthase 2
RNA: Ribonucleic acid
ROS: Reactive oxygen species
RT-PCR: Reverse transcription polymerase chain reaction
SDS: Sodium dodecyl sulfate
SEM: Standard error of the mean
SFA: Saturated fatty acid
SOD1: Superoxide dismutase1
SPP1: Secreted phosphoprotein 1
SREBPs: Sterol regulatory element-binding protein
SREBP1: Sterol regulatory element binding transcription factor 1
Th: Thompsons
THI: Temperature-humidity index
TLR4: Toll like receptor 4
TNFα: Tumor necrosis factor alpha
UPLC: Ultra Performance Liquid Chromatography
ULC/MC: Ultra Liquid Chromatography/Mass Spectrometry
UTX: Ubiquitously expressed prefoldin like chaperone
∆∆Ct: Delta-delta-cycle threshold
