# Supplementary material for: Maternal supplementation with n-3 fatty acids affects placental lipid metabolism, inflammation, oxidative stress, the endocannabinoid system, and the neonate cytokine concentrations in dairy cows
Source: J Anim Sci Biotechnol. 2024 May 21;15:74. doi: 10.1186/s40104-024-01033-4 (PMC11106909; doi:10.1186/s40104-024-01033-4)
Supplement: Supplementary file 1 — Additional file 1: Fig. S1. Schematic representation of the experimental procedures and analyses performed in this study. Fig. S2. Functional categorization of DAPs in IPA. Fig. S3. Top canonical pathways according to the differential proteome analysis in placenta FO vs. FLX. Fig. S4. Selected networks based on IPA analysis of in FO vs. CTL and FO vs. FLX. [file 40104_2024_1033_MOESM1_ESM.docx]

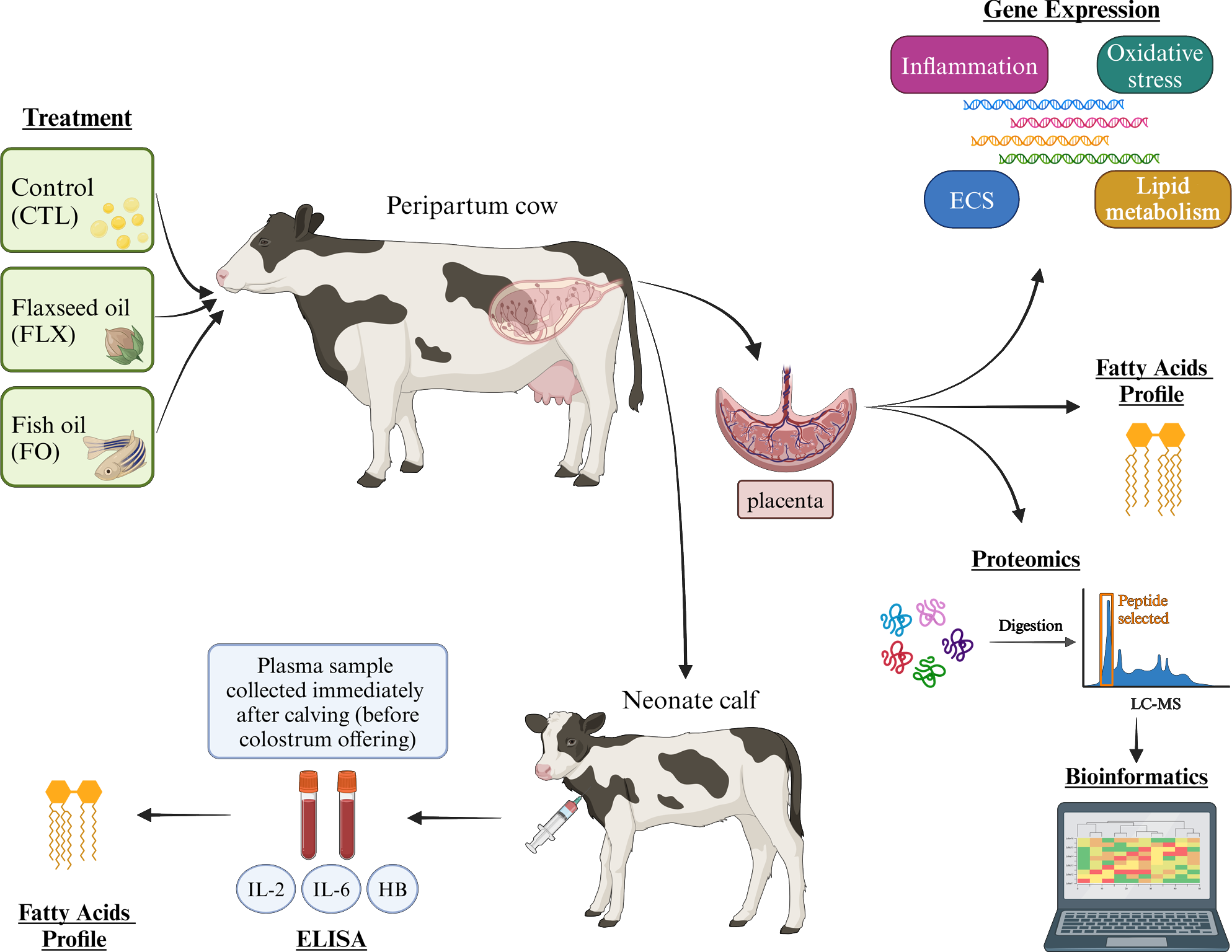
**Additional File 1: Supplementary Figures**

**Fig. S1** Schematic Representation of the experimental procedures and analyses performed in this study. Forty-two Israeli-Holstein dairy cows at d 257 of pregnancy were divided into three nutritional groups supplemented with (i) CTL – encapsulated saturated fat, (ii) FLX – encapsulated flaxseed oil providing ALA, or (iii) FO – encapsulated fish oil providing EPA and DHA. For each treatment, cotyledon samples were collected from placentas immediately after the delivery and analyzed for variations in gene expression, fatty acid profile, and proteomics. Blood samples were collected from calves immediately after birth (before colostrum intake) for fatty acid profile analysis and quantification of plasma concentrations of IL-2, IL-6 and haptoglobin. Image was generated using BioRender.com

###
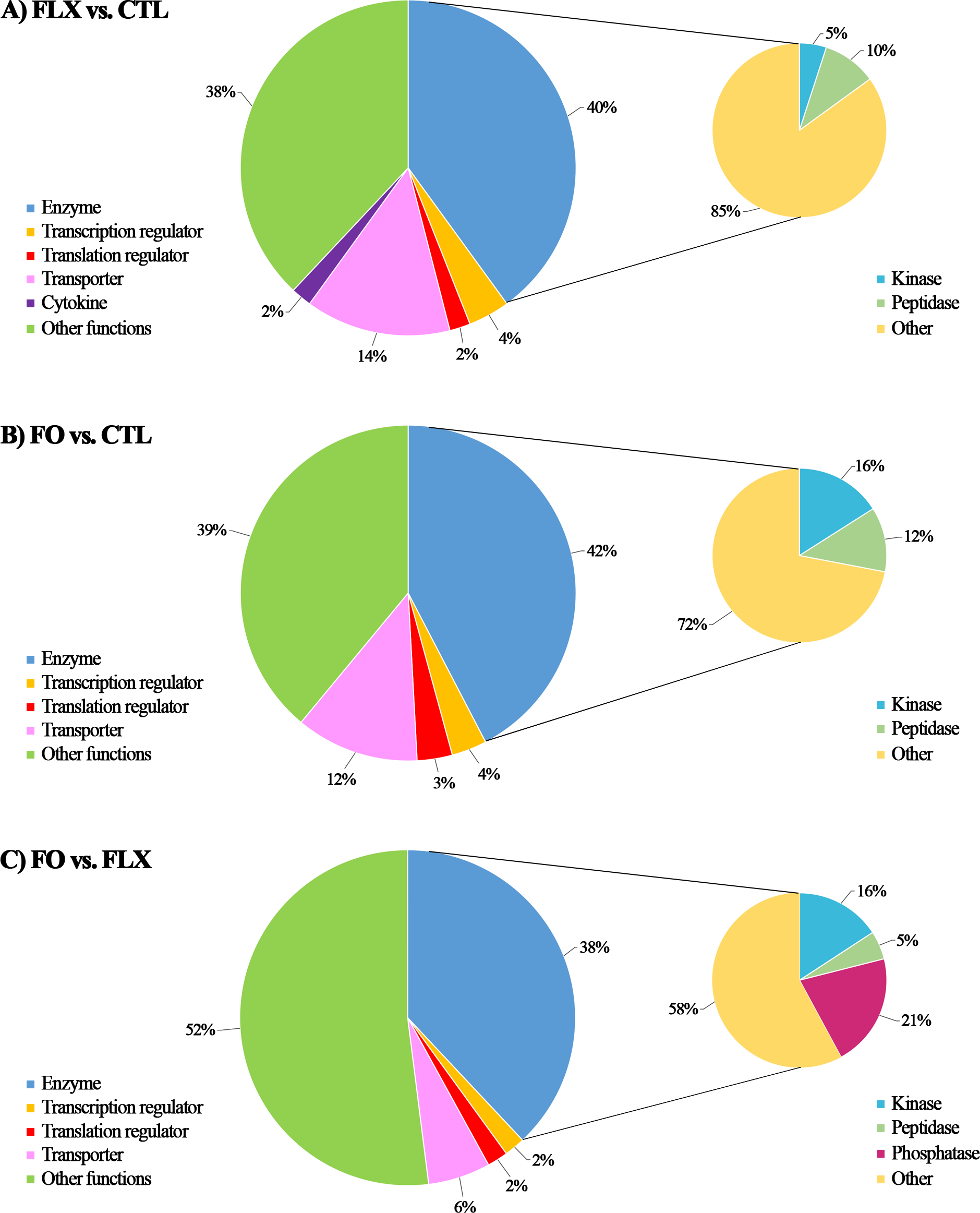
Fig. S2. Functional categorization of DAPs in IPA. Pie charts showing categories according to molecular function groups in FLX vs. CTL (A), FO vs. CTL (B), and FO vs. FLX (C)

###
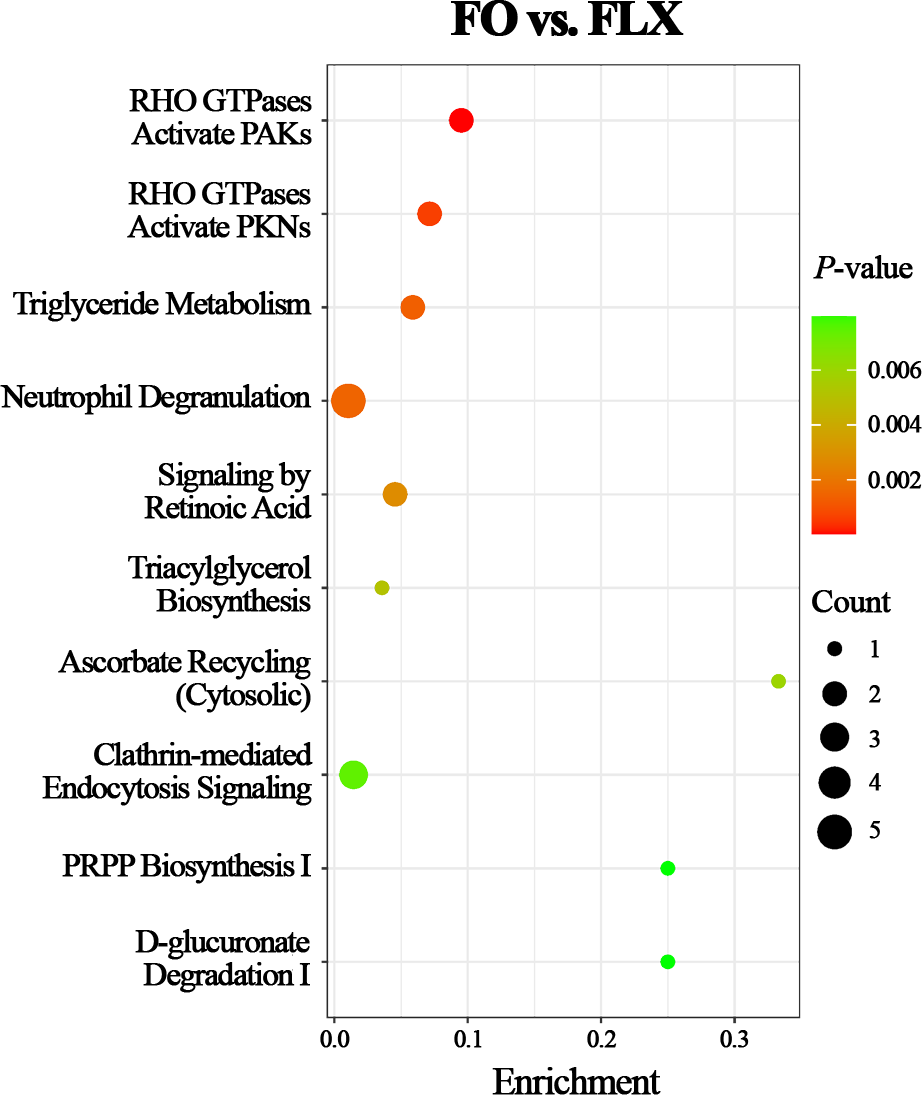
Fig. S3 Top canonical pathways according to the differential proteome analysis in placenta FO vs. FLX. Dairy cows supplemented pre-partum with (i) FO – encapsulated fish oil providing EPA and DHA, or (ii) FLX – encapsulated flaxseed oil providing ALA. Enrichment (*X*-axis) is calculated by dividing the number of DAPs (FC ± 1.5) assigned to a particular pathway by the total number of molecules within that pathway. *P*-value (threshold of ≤0.05) is depicted by color scale. Plot was generated using SRplot [41]

###
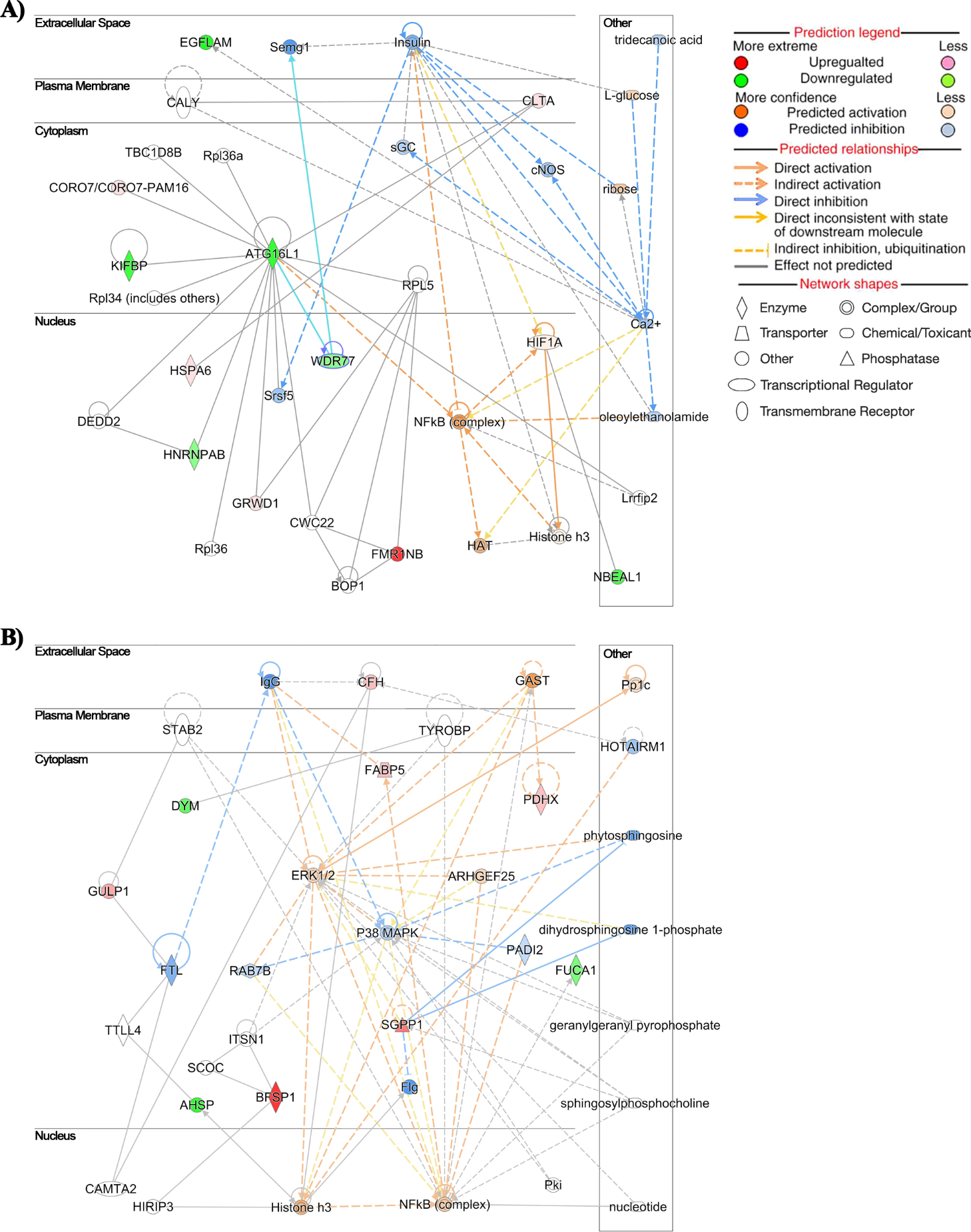
Fig. S4 Selected networks based on IPA analysis of in FO vs. CTL and FO vs. FLX. (A) FO vs. CTL: Network 'Cardiovascular System Development and Function, Organ Morphology, Organismal Development' includes the DAPs: *FABP3* heart-type fatty acid binding protein, *NOS2* Nitric oxide synthase, *PTGS2* Prostaglandin-endoperoxide synthase 2*.* (B) FO vs. FLX: Network 'Cellular Development, Dermatological Diseases and Conditions, Organismal Injury and Abnormalities' includes the DAPs: *FABP5* epidermal-type fatty acid binding protein, *NFkB-complex* NFKB2 protein, *P38 MAPK* p38 mitogen-activated protein kinase, and *CFH* complement factor H. Dairy cows at d 257 of pregnancy were divided into three nutritional groups supplemented with (i) CTL – encapsulated saturated fat, (ii) FLX – encapsulated flaxseed oil providing ALA, or (iii) FO – encapsulated fish oil providing EPA and DHA. Image was generated using [www.qiagen.com/ingenuity](http://www.qiagen.com/ingenuity)
