# Supplementary material for: Maternal supplementation with n-3 fatty acids affects placental lipid metabolism, inflammation, oxidative stress, the endocannabinoid system, and the neonate cytokine concentrations in dairy cows
Source: J Anim Sci Biotechnol. 2024 May 21;15:74. doi: 10.1186/s40104-024-01033-4 (PMC11106909; doi:10.1186/s40104-024-01033-4)
Supplement: Supplementary file 3 — Additional file 3. In-silico docking studies of bovine FABPs with selected endocannabinoids. Detailed description of the methods and results of the analysis. [file 40104_2024_1033_MOESM3_ESM.docx]

**Additional file 3**

# Methods

## **In silico prediction of FABP3, FABP5 and FABP7 structures by molecular modeling**

FABP3 (Accession number: P10790), FABP5 (Accession number: P55052) and FABP7 (Accession number: Q09139) sequences were collected from UNIPROT database using *Bos taurus* model for modelling the protein structures. The sequences were submitted to SMART ([SMART.embl.de](https://smart.embl.de/)) server in FASTA format for domain identification. The similar template was identified using BLAST server [1] to find out the related protein structure in PDB database using the FABP3, FABP5 and FABP7 domains. The FABP3, FABP5 and FABP7 were aligned with template sequences using the default parameters in ClustalX [2]. The structures were predicted by homology modeling method for FABP3, FABP5 and FABP7 using MODELLER9V7 software [3]. From the generated models, model with least modeller objective function was selected and further stabilized by molecular dynamics using NAMD 2.8 and CHARMM27 force filed. In the molecular dynamics studies, the Root Mean Square Deviation (RMSD) of FABP3, FABP5 and FABP7 were stabilized and to find the Stereochemical quality of protein structure, Ramachandran plot server was used in PROCHECK [2]. The environment profile is checked using structure evaluation server ERRAT [4].

## **Active site identification of target proteins**

After the final models were built, the possible binding sites of target proteins from *Bos taurus* were searched based on the structural comparison of template and the model build and also with CASTp server ([http://cast.engr.uic.edu/cast/](about:blank)). The active site of target proteins domain were predicted using SPDBV program based on the structure-structure comparison of template [3].

## **Docking studies of 2-AG and AEA compounds with target proteins**

The compounds (2-AG, AEA and DHEA) were docked to target proteins using GOLD 3.0.1 software, a genetic algorithm which uses strategy covering three genetic operators like migrations, mutations and cross overs [5]. The compounds docked into the active site of target proteins were thoroughly studied by molecular mechanics calculations. The parameters used to run Genetic algorithm in this study were pressure (1.1), population size (100), number of island (1), number of operations (10,000), and niche size. Parameters for migration, crossover and mutation were adjusted to 10, 100 and 100 respectively. The default algorithm speed was selected and the active site in target proteins was defined within a 10A° radius with the centroid as HH atom of 55 residue respectively. The best and most energetically favorable conformation of each compound was identified and selected after docking, each compound individual binding poses were studied and calculated the interactions with the protein.

## **GOLD score fitness function**

The four parameters, external vanderwaals energy, internal vanderwaals energy, external H-bond and internal hydrogen bond energy were considered for GOLD fitness function score. For predicting compound binding positions an empirical correction was done by multiplying external vanderwaals score with 1.375.

# Results

The FABP3, FABP5 and FABP7 sequences from *Bos taurus* collected from Uniprot database used to develop a three dimensional structures. The structures generated using NMR Study of Bovine Heart Fatty Acid Binding Protein (PDB ID:1BWY) from protein data bank as a result of BLAST search where the template showed maximum similarity. The alignment between these sequences showed conserved regions in both and the FABP3, FABP5 and FABP7 structure was optimized by molecular dynamics and validated using Ramachandran plot server using PROCHECK program (Fig. S1). The predicted FABP3, FABP5 and FABP7 structure showed 2 helices and 10 sheets in quaternary structures.

###
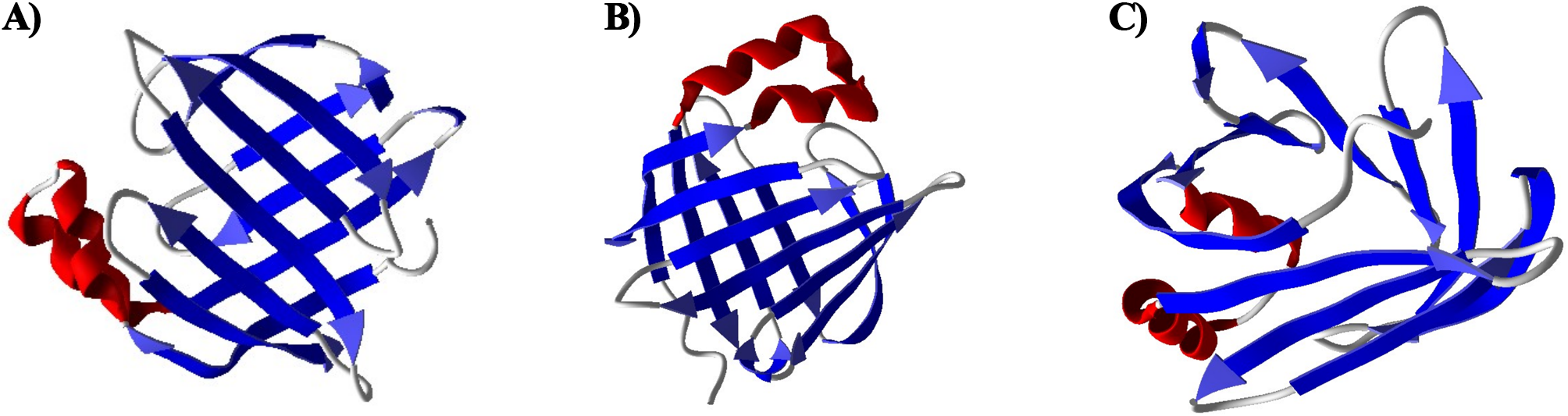
Fig. S1 Modeled structure of FABP3 (A), FABP5 (B) and FABP7 (C) with ten helices and two sheets

## **Active site identification of target proteins**

After structure validation studies, the possible binding sites of target protein was searched based on the structural comparison of template and the models build and also with CASTp server and the residues identified are shown in fig (Fig. S2). The compounds 2-AG, AEA, and DHEA were collected from Pubchem, designed using chemsketch software.

###
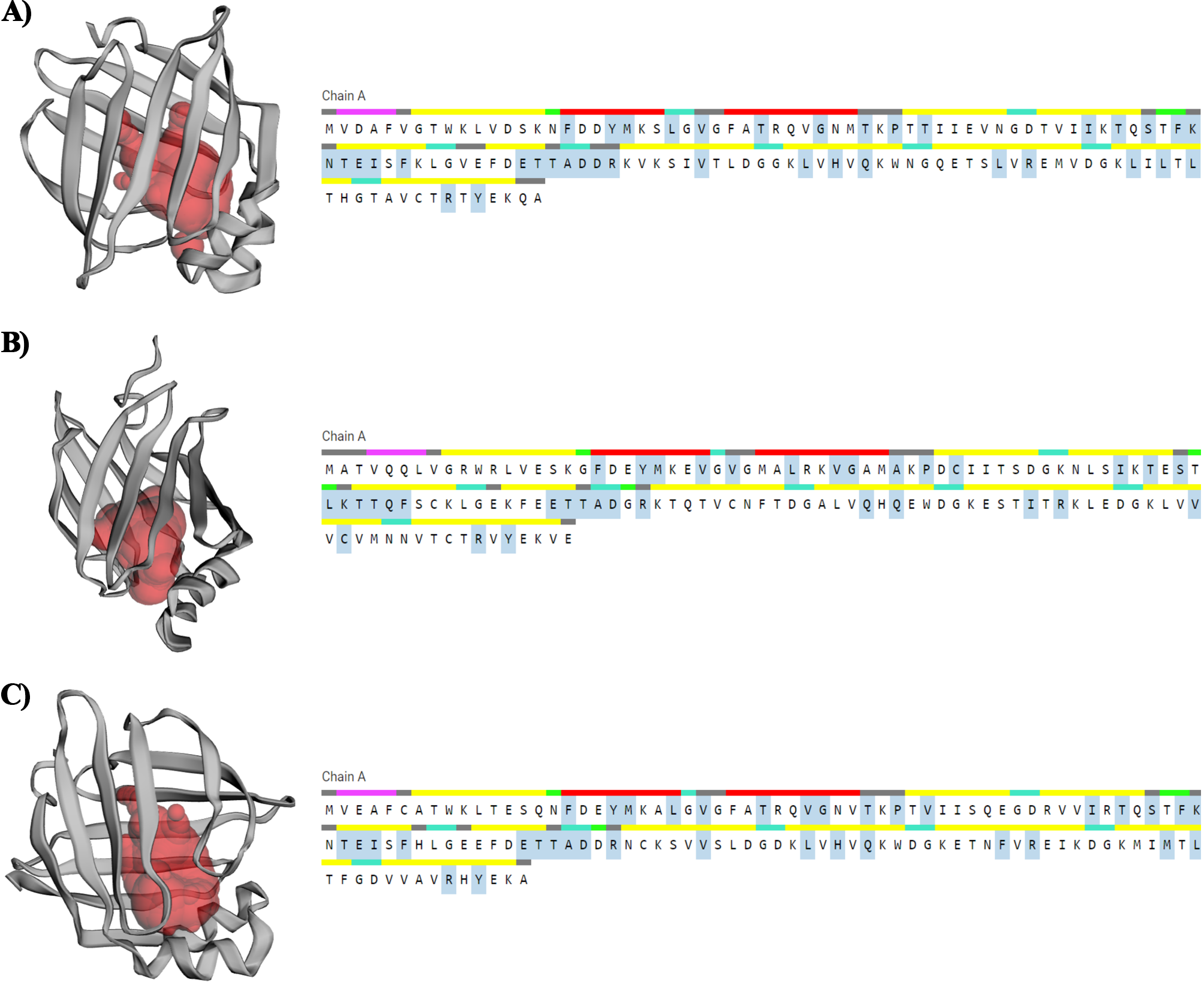
Fig. S2 The active site pockets of the Sterol-3-beta-glucosyltransferase showed area and volume (red color) and amino acids involved in active site of FABP3 (A), FABP5 (B) and FABP7 (C) from *Bos taurus*

## **Binding studies of compounds on target protein**


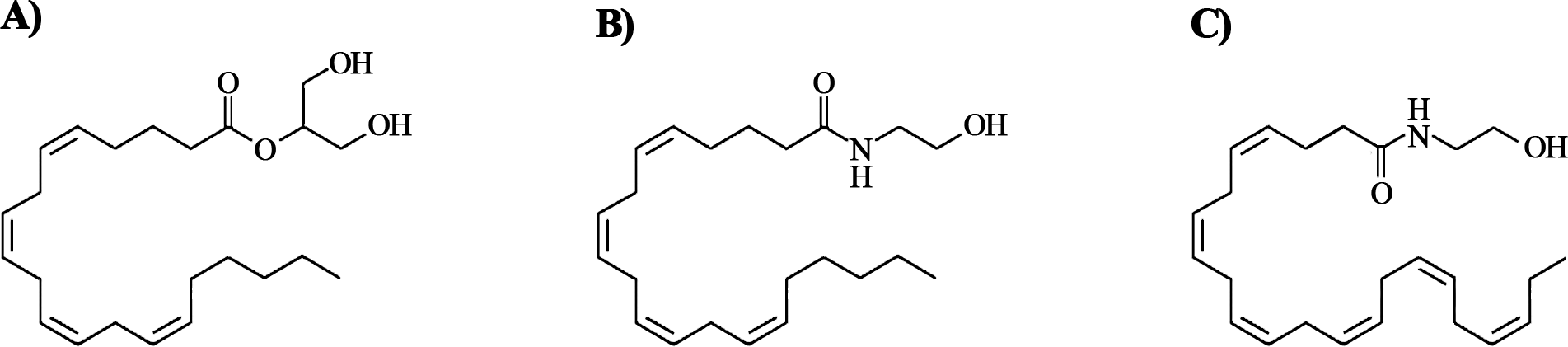
In this study, possible binding sites of target proteins were found using CASTp server. From these predicted binding sites, largest binding site in volume and size was selected and amino acids in this region were selected for docking studies. The 2-AG (Fig. S3A), AEA (Fig. S3B), and DHEA (Fig. S3C) were docked into target proteins using GOLD 3.0.1 and all docking solutions for target protein were ranked according to the GOLD fitness function.

### Fig. S3 Structure of 2-Arachidonoylglycerol (A), N-arachidonoylethanolamine (B) and Docosahexaenoyl Ethanolamide (C)

From the docking results it was confirmed that FABP3 binds 2-AG with good fitness score (Fig. S4). In binding studies, it was confirmed that 2-AG formed two hydrogen bonds with a bond length of 1.50A^o^, and 1.75A^o^ with FABP3. In FABP3-2-AG complex, hydrogen atom of 2-AG formed one hydrogen bond with oxygen atom (O4) of ASP3 and another hydrogen bond between hydrogen atom and oxygen atom (O3) of LYS131 with binding energy 6.35 kcal/mol.

From the docking results it was confirmed that FABP3 binds AEA inhibitor with good fitness score (Fig. 5A). In binding studies, it was confirmed that AEA formed two hydrogen bonds with a bond length of 1.25A^o^, and 1.13A^o^ with FABP3. In FABP3-AEA complex, hydrogen atom of AEA formed one hydrogen bond with oxygen atom (O4) of ASP3 and another hydrogen bond between hydrogen atom and oxygen atom (O2) of VAL45 with binding energy 4.16 kcal/mol.

### *
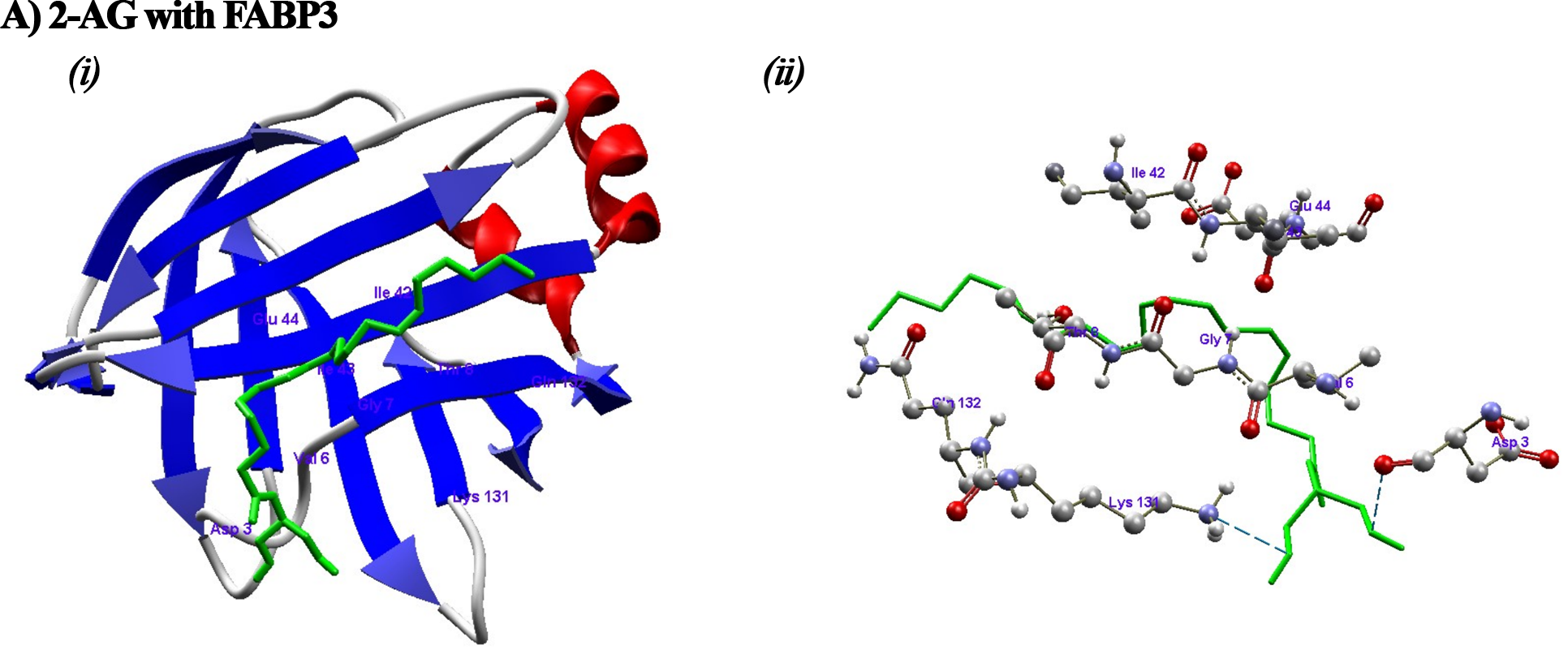
*Fig. S4: Binding studies of 2-AG and AEA with FABP3 from *Bos taurus*. (A*i*) Docking studies of 2-AG with FABP3; (A*ii*) Amino acids involved in hydrogen bonding with the 2-AG; hydrogen bonds were represented with blue dotted lines

From the docking results it was confirmed that FABP5 binds 2-AG with good fitness score (Fig. S5A). In binding studies, it was confirmed that 2-AG formed two hydrogen bonds with a bond length of 1.75A^o^, and 1.83A^o^ with FABP5. In FABP5-2-AG complex, two hydrogen atoms of 2-AG formed two hydrogen bonds with nitrogen atom (N2) of MET1 with binding energy 5.90 kcal/mol.

From the docking results it was confirmed that FABP5 binds AEA with good fitness score (Fig. S5B). In binding studies, it was confirmed that AEA formed one hydrogen bond with a bond length of 1.25A^o^ with FABP5. In FABP5-AEA complex, hydrogen atom of AEA formed one hydrogen bond with oxygen atom (O3) of ASP113 with binding energy 3.55 kcal/mol.

###
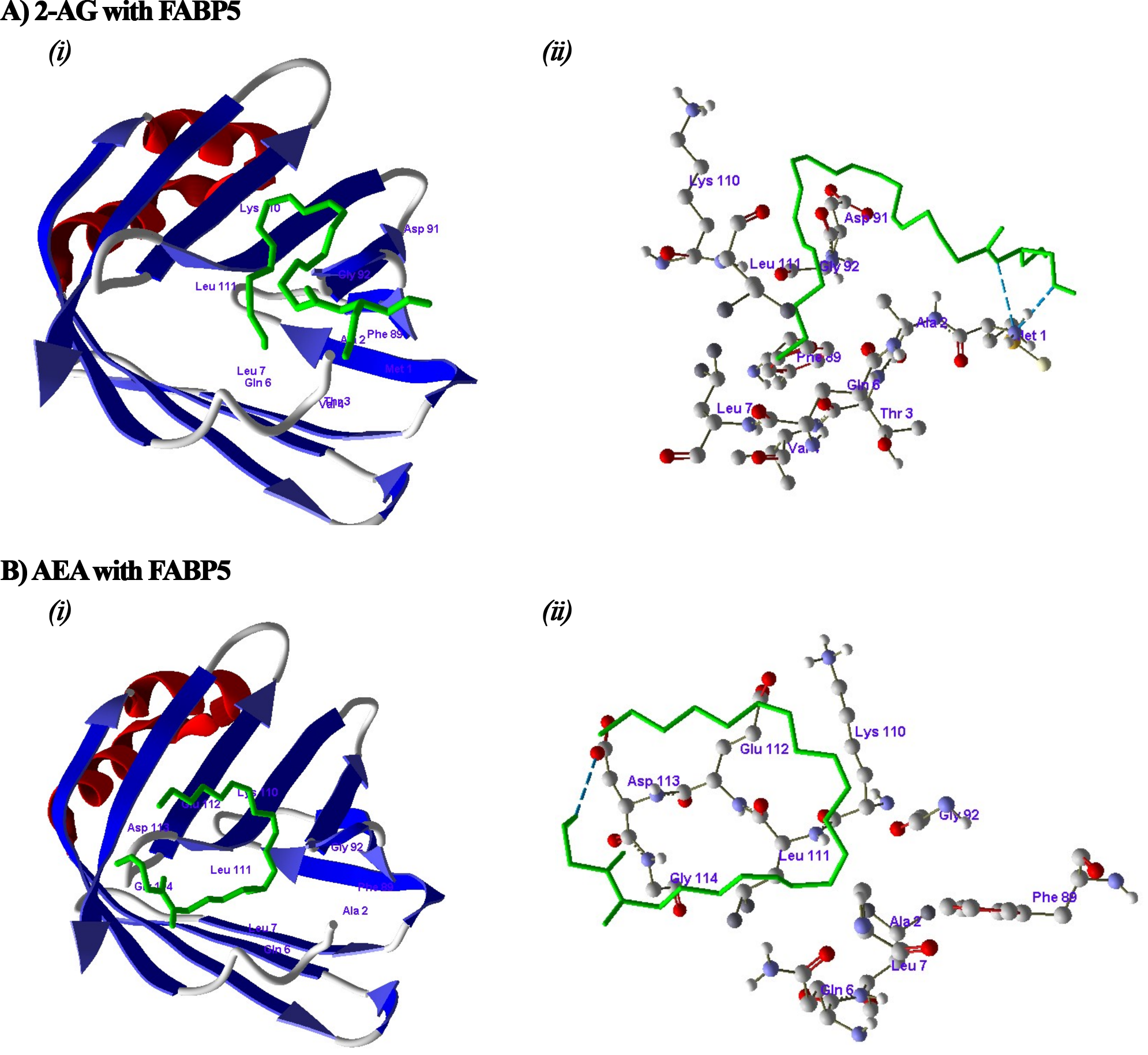
Fig. S5 Binding studies of 2-AG and AEA with FABP5 from *Bos taurus*. (A*i*) Docking studies of 2-AG with FABP5; (A*ii*) Amino acids involved in hydrogen bonding with the 2-AG; hydrogen bonds were represented with blue dotted lines. (B*i*) Docking studies of AEA with FABP5; (B*ii*) Amino acids involved in hydrogen bonding with the AEA; hydrogen bonds were represented with blue dotted lines

###
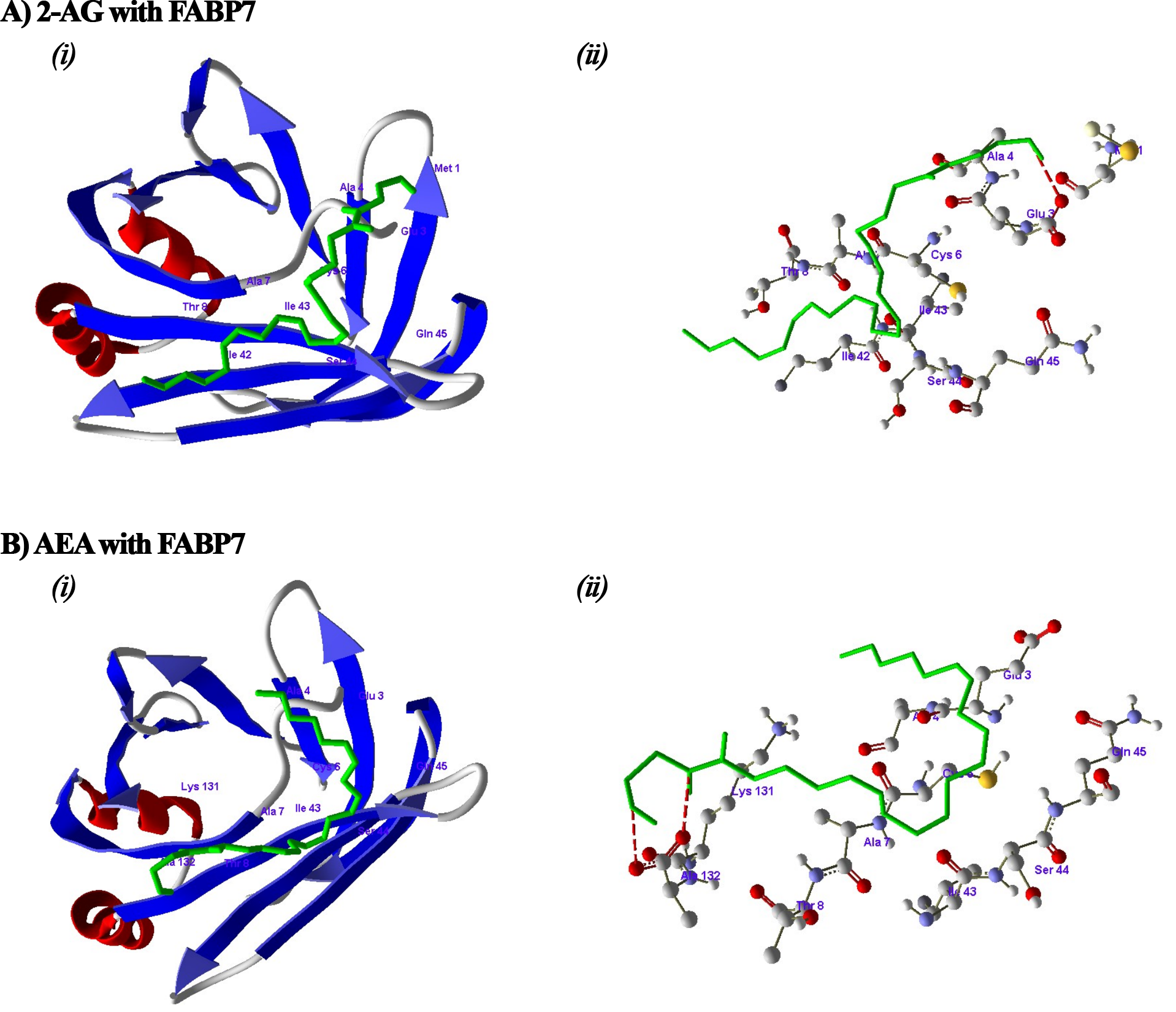
Fig. S6 Binding studies of 2-AG and AEA with FABP7 from *Bos taurus*. (A*i*) Docking studies of 2-AG with FABP5; (A*ii*) Amino acids involved in hydrogen bonding with the 2-AG; hydrogen bonds were represented with blue dotted lines. (B*i*) Docking studies of AEA with FABP7; (B*ii*) Amino acids involved in hydrogen bonding with the AEA; hydrogen bonds were represented with red dotted lines

From the docking results it was confirmed that FABP7 binding with 2-AG has low fitness score (Fig. S6A). In binding studies, it was confirmed that 2-AG formed one hydrogen bond with a bond length of 1.10A^o^ with FABP7. In FABP7-2-AG complex, hydrogen atom of 2-AG formed one hydrogen bond with oxygen atom (O2) of GLU3 with binding energy 0.06 kcal/mol.

From the docking results it was confirmed that FABP7 binding AEA has low fitness score (Fig. S6B). In binding studies, it was confirmed that AEA formed one hydrogen bond with a bond length of 1.08A^o^, with FABP7. In FABP7-AEA complex, hydrogen atom of AEA formed one hydrogen bond with oxygen atom (O2) of ALA132 with binding energy 0.15 kcal/mol.

**DHEA docking with FABP7**

From the docking results it was confirmed that FABP7 binds DHEA with high fitness score (Fig. 5B). In binding studies, it was confirmed that DHA formed three hydrogen bonds with a bond length of 1.25 A^o^, 1.50 A^o^ and 1.15A^o^ with FABP7. In FABP7-DHA complex, Oxygen atom (O2) of DHA formed two hydrogen bonds with oxygen atom (O6), and nitrogen (N3) of GLN45 and one hydrogen bond with oxygen atom (O2) of GLU3 with binding energy 10.43 kcal/mol.

# References

1. Ben Meir YA, Daddam JR, Kra G, Kamer H, Portnick Y, Levin Y, et al. Proteomic analysis of adipose tissue revealing differentially abundant proteins in highly efficient mid-lactating dairy cows. Sci Rep. 2022;12:9721.

2. Daddam JR, Sreenivasulu B, Peddanna K, Umamahesh K. Designing, docking and molecular dynamics simulation studies of novel cloperastine analogues as anti-allergic agents: Homology modeling and active site prediction for the human histamine H1 receptor. RSC Adv. 2020;10:4745–54.

3. Lin YM, Badrealam KF, Kuo CH, Daddamc J, Asokanc Shibu M, Lin KH, et al. Small Molecule Compound Nerolidol attenuates Hypertension induced hypertrophy in spontaneously hypertensive rats through modulation of Mel-18-IGF-IIR signalling. Phytomedicine. 2021;84:153450.

4. Daddam JR, Dowlathabad MR, Panthangi S, Jasti P. Molecular docking and P-glycoprotein inhibitory activity of Flavonoids. Interdiscip Sci – Comput Life Sci. 2014;6:167–75.

5. Kra G, Daddam JR, Moallem U, Kamer H, Ahmad M, Nemirovski A, et al. Effects of environmental heat load on endocannabinoid system components in adipose tissue of high yielding dairy cows. Animals. 2022;12(6):795.
